# Multilocus sequence based identification and adaptational strategies of *Pseudomonas* sp. from the supraglacial site of Sikkim Himalaya

**DOI:** 10.1371/journal.pone.0261178

**Published:** 2022-01-24

**Authors:** Srijana Mukhia, Anil Kumar, Poonam Kumari, Rakshak Kumar, Sanjay Kumar

**Affiliations:** 1 Biotechnology Division, CSIR-Institute of Himalayan Bioresource Technology, Palampur, Himachal Pradesh, India; 2 Department of Microbiology, Guru Nanak Dev University, Amritsar, Punjab, India; 3 Academy of Scientific and Innovative Research (AcSIR), CSIR- Human Resource Development Centre (CSIR-HRDC), Ghaziabad, Uttar Pradesh, India; Hokkaido University, JAPAN

## Abstract

Microorganisms inhabiting the supraglacial ice are biotechnologically significant as they are equipped with unique adaptive features in response to extreme environmental conditions of high ultraviolet radiations and frequent freeze-thaw. In the current study, we obtained eleven strains of *Pseudomonas* from the East Rathong supraglacial site in Sikkim Himalaya that showed taxonomic ambiguity in terms of species affiliation. Being one of the most complex and diverse genera, deciphering the correct taxonomy of *Pseudomonas* species has always been challenging. So, we conducted multilocus sequence analysis (MLSA) using five housekeeping genes, which concluded the taxonomic assignment of these strains to *Pseudomonas antarctica*. This was further supported by the lesser mean genetic distances with *P*. *antarctica* (0.73%) compared to *P*. *fluorescens* (3.65%), and highest ANI value of ~99 and dDDH value of 91.2 of the representative strains with *P*. *antarctica* PAMC 27494. We examined the multi-tolerance abilities of these eleven *Pseudomonas* strains. Indeed the studied strains displayed significant tolerance to freezing for 96 hours compared to the mesophilic control strain, while except for four strains, seven strains exhibited noteworthy tolerance to UV-C radiations. The genome-based findings revealed many cold and radiation resistance-associated genes that supported the physiological findings. Further, the bacterial strains produced two or more cold-active enzymes in plate-based assays. Owing to the polyadaptational attributes, the strains ERGC3:01 and ERGC3:05 could be most promising for bioprospection.

## Introduction

Glacier ice has recently gained recognition as a biome driven exclusively by microorganisms [1, 2]. Encompassing the topmost layer of ice, the supraglacial zone receives ample sunlight and is subjected to the deposition of microbial cells plus nutrients chiefly by the wind [3]. Moreover, the liquid water produced during the surface melting of ice promotes and supports a rich microbial life on the glacial surfaces [3, 4]. Bacteria inhabiting the supraglacial site are bestowed with special adaptive features as they are constantly subjected to subfreezing temperatures, high hydrostatic pressure, low nutrient availability, and high exposure to ultraviolet radiations [5]. The mechanisms of their survival include the production of useful biomolecules in the form of cold-active enzymes and ice-binding proteins for maintaining the molecular central dogma and membrane fluidity at freezing conditions [6, 7]. Cold-active proteases from microorganisms isolated from cold habitats represent a major group of enzymes essential for the metabolic and physiological functioning of an organism [6]. Another cellular protective response involves the production of photoprotective compounds that respond to osmotic and oxidative stress resulting from high radiation exposure [7, 8].

Among bacterial communities, the members of the genus *Pseudomonas* are known for their metabolically versatile nature and occurrence in diverse ecological niches [9]. The taxonomy of this genus has been revised from time to time and is now classified into two major lineages: *P*. *aeruginosa* group that includes all the clinical isolates, and *P*. *fluorescens* complex containing the environmental isolates, which are further subdivided into nine subgroups [10]. The *P*. *fluorescens* complex forms a relatively diverse group whose vast genetic and phenotypic heterogeneity poses complexity in their taxonomic assessment. A tenth subgroup has been added within the *P*. *fluorescens* complex named *P*. *antarctica* that consists of Antarctic species *P*. *antarctica* PAMC 27494 and *P*. *extremaustralis* 14–3 [9]. These species were previously placed under the *P*. *fluorescens* subgroup. So, there lies a narrow line between the subgroups *P*. *antarctica* and *P*. *fluorescens*.

We have been classifying the psychrotrophic bacteria from the East Rathong glacier of Sikkim Himalaya to maintain its repository of genomic data [7, 11–16], as well as to study its bioprospection potential [17, 18]. While exploring the supraglacial site, we observed taxonomic ambiguity among a dominant group of *Pseudomonas* sp. We obtained 11 unique morphotypes with distinct physiological characteristics, showing the closest 16S rRNA gene sequence similarity to *P*. *fluorescens* DSM 50090^T^ (S1 Table), but further evaluation of its taxonomic position with phylogenetic clustering based on the 16S rRNA gene sequence revealed taxonomic ambiguity.

Although 16S rRNA gene analysis is the best method with the largest database for bacterial taxonomic resolution, it is insufficient for efficient discrimination of bacterial taxa at the species level [7, 19]. Due to the extremely slow rate of evolution of the 16S rRNA gene, it often does not lead to the proper resolution of closely related species. On the contrary, the evolution of protein-encoding housekeeping genes is faster and provides a better solution for discriminating closely related *Pseudomonas* species [20]. Multilocus sequence analysis (MLSA) based on the combination of multiple housekeeping genes has been a reliable and preferred method for establishing the taxonomy at the species level [19].

Considering the significance of such bacteria from physical extremes of temperature and radiation, we conducted MLSA of eleven strains of *Pseudomonas* genus obtained from the supraglacial site of East Rathong glacier to resolve its taxonomy. To check their adaptive features and to establish the fact that this unique group of bacteria is the inhabitants of the pristine supraglacial environment, we investigated their survivability in physical conditions of freezing and UV radiation and examined their ability to produce extracellular cold-active enzymes. Further, we performed whole genome analysis of two representative strains for taxonomic validation and understanding genetic basis of high-altitude adaptation.

## Methods

### Sampling site

**Fig 1:**
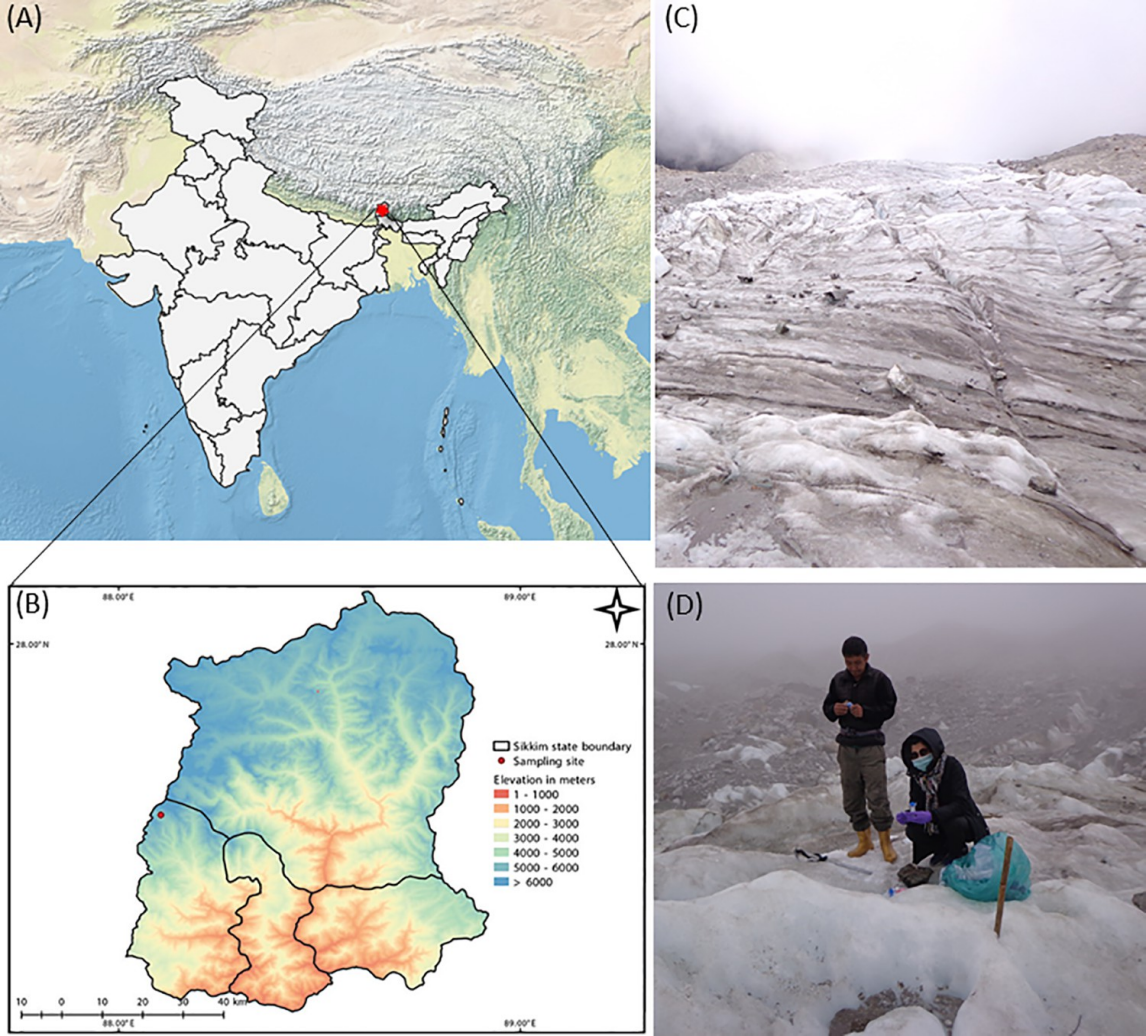
Map illustrating (A-B) geographical location of the sample collection site in Sikkim Himalaya, India. The red circle depicts the specific spot of the collected ice meltwater sample; (C-D) sampling site comprising the supraglacial site in the ablation zone of the East Rathong glacier. Made with Natural Earth, free vector, and raster map data @ naturalearthdata.com. The map was made in QGIS version 3.0.0 (URL: http://qgis.org). [The individuals in the manuscript Fig 1(D) have given written informed consent (as outlined in PLOS consent form) to publish the image].

Ice meltwater samples were collected from three points on the supraglacial site of the ablation zone of East Rathong glacier (Fig 1), located between latitudes 27°33’36"N and 27°36’40"N, longitudes 88°06’03"E and 88°07’38"E [21]. It is a south-east-facing, debris-free, and summer-nourished glacier in the West district of Sikkim that forms the dominant glacier in the Eastern Himalayas. The area experiences a cool and wet climatic condition, and snowfall is not unusual even during the monsoon season [22]. The sample collection was done in May with the forest permit obtained from the Forest and Environment Department, Government of Sikkim. Ice meltwater samples were collected in sterile 250 ml amber wide-mouth bottles (Tarson, India) and transported in ice buckets with ice packs. Samples were stored at 4°C until analysis.

### Isolation and identification of bacteria

The ice meltwater and the water samples of the glacier surface were enriched in sterile distilled water in shaking conditions for 2 h at 10°C. Subsequently, tenfold serial dilutions of each samples were made to plate in triplicate on R2A agar medium (pH 7.0; Himedia, India) and Antarctic Bacterial Medium (ABM) plates [peptone (0.5%, w/v), yeast extract (0.2%, w/v) and agar (2%, w/v)] and incubated at 10°C for 10–15 days. Viable bacteria obtained in the agar plates were counted as colony forming units (CFU). Unique morphotypes from each plate were purified and maintained on ABM plates. The pure cultures obtained were preserved using 20% glycerol at -80°C for further studies. Growth parameters were checked at different temperatures (4, 20, 28, 37, 50°C), pH (1, 3, 5, 7, 9, 11, 13) and varying salt concentrations (1–6%) in ABM agar plates.

### Molecular characterization

#### 16S rRNA gene sequencing and phylogenetic analysis

Genomic DNA was extracted from each isolate using the CTAB method, as given by Chen and Kuo [23]. The 16S rRNA gene was amplified by PCR using universal primers 27F (5’-AGAGTTTGATCCTGGCTCAG-3’) and 1492R (5’-GGTTACCTTGTTACGACTT-3’) [24]. PCR was performed with 20μl reaction mixtures containing approximately 50 ng of template DNA, 2 μM forward primer, 2 μM reverse primer, and 1X GoTaq Green PCR Master Mix (Promega, US). DNA amplification was carried out in G-Storm Thermocycler (Somerset, United Kingdom) with an initial denaturation step of 94°C for 5 min, followed by 30 cycles of denaturation at 94°C for 1 min, annealing at 55°C for 1 min, and extension at 72°C for 2 min and then a final extension step of 72°C for 5 min. The PCR products were purified prior to sequencing by treatment of ExoSAP-IT solution as per the manufacturer’s instructions (Affymetrix, US).

Purified PCR products were subjected to cycle sequencing using the forward, reverse, and internal primers with Big Dye Terminator cycle sequencing kit v.3.1 (Applied Biosystems, US) protocol as described earlier [25]. The sequencing reaction was performed with a 5 μL reaction mixture containing approximately 50 ng template DNA and 1 pmol of sequencing primers. Post reaction cleanup was performed using a Montage Sequencing Reaction cleanup kit (Millipore, US) using a Vacuum Pump Assembly (Millipore, US). Cleaned samples after cycle sequencing were bi-directionally sequenced using an automated Genetic Analyzer ABI 3130XL (Applied Biosystems, US).

The generated sequences were used to perform Basic Local Alignment Search Tool (BLAST) [26] analysis to determine the nearest phylogenetic neighbours against the NCBI GenBank database of validly published prokaryotic names (http://www.ncbi.nlm.nih.gov/genbank/). For phylogenetic analysis, 16S rRNA gene sequences of the neighbors were obtained from the GenBank database (NCBI), and Molecular Evolutionary Genetics Analysis software (MEGA version X) was used for phylogenetic analyses [27]. The sequences of identified phylogenetic neighbors were aligned using Clustal W inbuilt with MEGA X. *E*. *coli* K12 MG1655 was used as the outgroup organism. Maximum likelihood method using the Kimura 2-parameter model was employed to construct the phylogenetic trees with 1000 bootstrap replications to assess nodal support in the tree.

#### Genotyping by ERIC-PCR

The isolates that showed similar phenotypes were compared through enterobacterial repetitive intragenic consensus (ERIC)-PCR. Briefly, genomic DNA was extracted and subjected to PCR amplification using the primers ERIC1 (5’-ATGTAAGCTCCTGGGGATTCAC-3’) and ERIC2 (5’-AAGTAAGTACTGGGGTGAGCG-3’), as given by Khosravi et al. [28]. The thermocycling conditions consisted of first denaturation cycle at 95°C for 7 min followed by 30 cycles, including denaturation at 94°C for 1 min, annealing for 1 min at 52°C for ERIC-PCR, extension at 65°C for 8 min, one final extension cycle at 65°C for 16 min, and hold at 4°C. The amplified products were subjected to electrophoresis on 1.5% agarose gel, stained with 0.5 μg/μl ethidium bromide (Qiagen, Germany), and analyzed under UV light in a gel documentation system (Syngene G-BOX transilluminator, US). The banding profiles were observed. The visible bands were converted into a binary matrix and used to construct a dendrogram using the Jaccard similarity index and the unweighted pair group method (UPGMA) with the aid of software Past, version 3.25.

#### Multilocus sequence analysis (MLSA)

The eleven strains belonging to the species of *Pseudomonas* were studied for their phylogenetic relationship by multilocus sequence analysis of five housekeeping genes. In the current study, we used five housekeeping genes for MLSA of 11 *Pseudomonas* strains, *viz*. DNA gyrase subunit B (*gyrB*), which is a subunit of an important bacterial enzyme catalyzing the ATP-dependent negative super-coiling of circular double-stranded DNA; isoleucyl-tRNA synthetase (*ileS*), whose function is to charge tRNA^Ile^ with isoleucine; NADH dehydrogenase subunit D (*nuoD*), that plays an important role in the central metabolism of electron transport system; recombinase A (*recA*), linked with the homologous recombination in bacteria; and RNA polymerase sigma factor (*rpoD*), a major sigma factor involved in bacterial RNA transcription. These genes were selected based on the criteria that they are present as single copies in the genome and are homologous and ubiquitous in the studied taxa [19]. The primers used for PCR amplification of the housekeeping genes were based on previous studies [29] (S2 Table). The PCR condition for all genes consisted of: an initial denaturation step of 2 min at 94°C, followed by 35 cycles of 30s at 94°C, 20 s at 54°C, and 2 min at 72°C, and a final extension of 7 min at 72°C. After Sanger sequencing, the partial sequences obtained for these five genes were submitted in GenBank (NCBI). Based on 16S rRNA gene similarity, seventeen closely related *Pseudomonas* species were taken, and the partial sequences of these five housekeeping genes were retrieved from the complete genomes of corresponding type strains of *Pseudomonas* from the GenBank database. Multiple sequence alignment of the nucleotides was performed with CLUSTAL W, and the sequences were trimmed manually for subsequent phylogenetic analyses. Sequences were translated to the amino acid, and the open reading frame was determined in MEGA X. For phylogenetic analysis, the partial sequences of the five protein-coding genes were concatenated into a single alignment using MEGA X in the order: *gyrB-ileS-nuoD-recA-rpoD*. Partitionfinder v2.1.1 [30] was used to determine the best-fit partitioning schemes and substitution models of molecular evolution. Maximum likelihood (ML) trees were constructed with the same partitioning schemes and model using RAxMLGUI v1.5 [31] through the CIPRES Science Gateway for individual and concatenated datasets [32].

Kimura-2-Parameter (K2P) genetic distances [33] were calculated between the eleven *Pseudomonas* strains and their nearest neighbors taken from the NCBI database using MEGA, Version X.

#### Nucleotide polymorphism

Gene parameters, such as GC content, number of polymorphic sites, parsimony-informative sites, synonymous and non-synonymous sites, percentages of mean sequence similarities, number of nucleotide differences per site (Θ), nucleotide diversity per site (π), and Tajima’s D statistic for individual gene sequences and the concatenated sequence were computed using MEGA X. The confidence of the branches of the ML tree was based on 1000 bootstrap replicates. *E*. *coli* K12 MG1655 was used as an outgroup.

### Physiological characterization

#### Tolerance to UV-C

For the radiation resistance test, the colony count method corresponding to UV-C irradiated aliquots and non-irradiated control was performed as described previously [34], with minor modifications. The bacterial strains were grown in ABM broth till cell O.D. 600 of ~1.0 was attained. After centrifugation at 8,000 rpm for 5 min, the pellet was washed and re-suspended in normal saline. UV-C exposures of 150, 300, and 450 Jm^-2^ were given to the cell suspensions. Serial dilutions of UV irradiated as well as non-irradiated bacterial cultures were made and spread plated. After incubation for 3–5 days at 20°C, the number of colony-forming units was determined, and the survival percentage was calculated. For comparison, a mesophilic type strain *Pseudomonas aeruginosa* MTCC 2453 and a radioresistant type strain *Deinococcus radiodurans* MTCC 4465 were subjected to the same conditions of UV-C exposure.

#### Tolerance to freezing

Tolerance to freezing was checked for 96 h. Cultures were grown till the stationary phase in the ABM broth, and culture tubes in triplicates were placed in a -20°C freezer. After every 24 h of freezing, tubes were removed, thawed for 1 h at 20°C, and 100 μL of the thawed culture was serially diluted in normal saline. The diluted culture was spread on ABM agar and incubated at 20°C for 2–4 days. The average of the triplicate colony counts was used for determining the survival percentage. Plates of unfrozen culture served as 0-time point control, and a mesophilic type strain *Pseudomonas aeruginosa* MTCC 2453 was subjected to the same freezing condition.

#### Estimation of cold-active enzymes

The isolates were screened for the production of hydrolytic enzymes such as amylase, cellulase, lipase, and protease at 10°C by spotting the cultures on their selective media containing a specific substrate. Protease activity was assayed in skim-milk agar. A clear zone around the colony indicated the production of proteases [35]. Lipase activity was checked on the enrichment medium tributyrin agar. The colonies with clear hydrolysis zones were marked as lipase producers [36]. Carboxymethyl cellulose (CMC) agar was used for the detection of cellulase activity. On flooding the plates with Gram’s iodine, the zone of clearance around the bacterial colonies represented positive cellulase producers [37]. Amylase activity was checked on starch agar plates. Amylolytic isolates were selected by flooding the starch agar plates with Gram’s iodine solution. Isolates with distinct, clear zone around the colonies were identified as amylase producers [38].

Extracellular protease activities of eleven bacterial isolates were determined by a previously described method [39]. In brief, eleven bacterial isolates were grown in Antarctic Bacterial Medium (0.2% yeast extract, 0.5% peptone) broth for 12 h at 20°C. The bacterial growth was checked at 600 nm. 1% (v/v) bacterial culture was seeded into the protease production medium (K_2_HPO_4_ 0.1%, KH_2_PO_4_ 0.05%, CaCl_2_ 0.02%, MgSO_4_.7H_2_O 0.05%, Glucose 1%) with 1% skim milk as substrate and incubated at 20°C, 120 rpm. After 48 h of incubation, the cultures were centrifuged at 10,000 rpm for 15 min at 4°C. The cell-free supernatant was used as a source of enzyme. For the enzymatic assay, the reaction mixture containing 100 μl of the enzyme and 400 μl of 1% casein solution in 50 mM Tris buffer (pH 8) was incubated for 10 min at 5 and 15°C. The reaction was terminated by adding 0.5 ml of trichloroacetic acid (1.2 M) and centrifuged for 10 min at 6000xg. 500 μl of the filtrate was mixed with 1 ml of 400 mM Na_2_CO_3_ solution and 50 μl Folin- Ciocalteu’s reagent. The amount of tyrosine released was determined spectrophotometrically at 660 nm against the enzyme blank. The control was treated in the same way, except TCA was added before enzyme addition. One unit of protease activity was equivalent to the amount of enzyme that required releasing 1 μg of tyrosine/ml/min under standard assay conditions.

### Genome sequencing, annotation, and analysis of representative strains

Two potential bacterial strains ERGC3:01 and ERGC3:05, were proceeded for whole-genome sequencing after checking the purity of the extracted DNA using Qubit® 2.0 Fluorometer. DNA library was prepared according to the SQK-LSK109 Ligation Sequencing Kit protocol (Oxford Nanopore Technologies, Oxford, UK). The prepared library was loaded onto the flow cell (FLO-MIN106; R9.4 chemistry) and sequenced using MinION controlled by MinKNOW software. The raw nanopore reads were basecalled using guppy basecaller v5.0.11, quality trimmed, and after adapter removal by Porechop v0.2.4, the processed reads were assembled *de novo* using Flye assembler v2.8.3 [40]. The Flye generated draft assembly was polished with two rounds of Racon [41] and further by the Medaka tool (https://nanoporetech.github.io/medaka/). The final genome assembly was checked for completeness using BUSCO v5.2.2 [42] and CheckM v1.1.3 [43]. The assembled genome was checked for the presence of plasmid using an online PlasmidFinder 2.1 tool (https://cge.cbs.dtu.dk/services/PlasmidFinder/) and annotated by NCBI PGAP pipeline v 5.2 [44]. The final genome assembly was uploaded to the online Type Strain Genome Server (TYGS) tool [45] to find the nearest bacterial strains. A phylogenetic tree was constructed between the nearest neighbours based on 91 core genes using the UBCG pipeline [46]. Based on the phylogeny obtained from the UBCG pipeline, the average nucleotide identity (ANI) value and genome–genome distance of the studied strains were calculated by comparing assembled genomes with publicly available *Pseudomonas* genomes using OrthoANI tool (EzBioCloud) [47] and digital DNA–DNA hybridisation (dDDH) tool (http://ggdc.dsmz.de/).

### Statistical analysis

All experiments were conducted in triplicate, and the results were presented as the mean ± standard deviation. Statistical analysis for significant differences was performed by one-way ANOVA followed by Dunnett’s multiple comparison test (at *p* < 0.05 statistically significant differences) using the software GraphPad Prism7 (GraphPad Software, Inc., San Diego, USA).

### Nucleotide sequence accessions numbers

The accession numbers obtained from NCBI GenBank for the 16S rRNA gene sequences against each strain are provided in S1 Table, and those for partial housekeeping gene sequences lie from MN238515-MN238591. The genome sequences of *Pseudomonas* sp. ERGC3:01 and ERGC3:05 have been deposited at DDBJ/EMBL/GenBank under the accession numbers JAIEWI000000000 and CP080597, respectively.

## Results

### Isolation, physiological characterization, and taxonomic affiliation of bacteria

The pH of the collected ice meltwater ranged from 6 to 6.7 at the collection site, while the temperature was noted 8°C at the laboratory before further processing of the samples (S3 Table). The cultivated bacteria with unique morphotypes were selected. The eleven strains, reported in the current study, showed white to creamish pigmentation and produced fluorescent pigment on King’s B medium [48] (S1 Fig). The physiological characteristics of these eleven strains were evaluated (S1 Table). The strains exhibited growth in the temperature range of 4–28°C, indicating their psychrotolerant nature. Five of the strains could grow till 37°C, the temperature optima for all being 20°C. Among eleven, ten strains grew over a pH range of 4–12, while one strain showed growth in the pH range of 5–12. Most strains showed tolerance to NaCl concentration ranging from 1–4% (w/v), while ERCE:11 could only tolerate up to 3% NaCl, and ERGC9:06 was tolerant to 5% NaCl.

**Fig 2:**
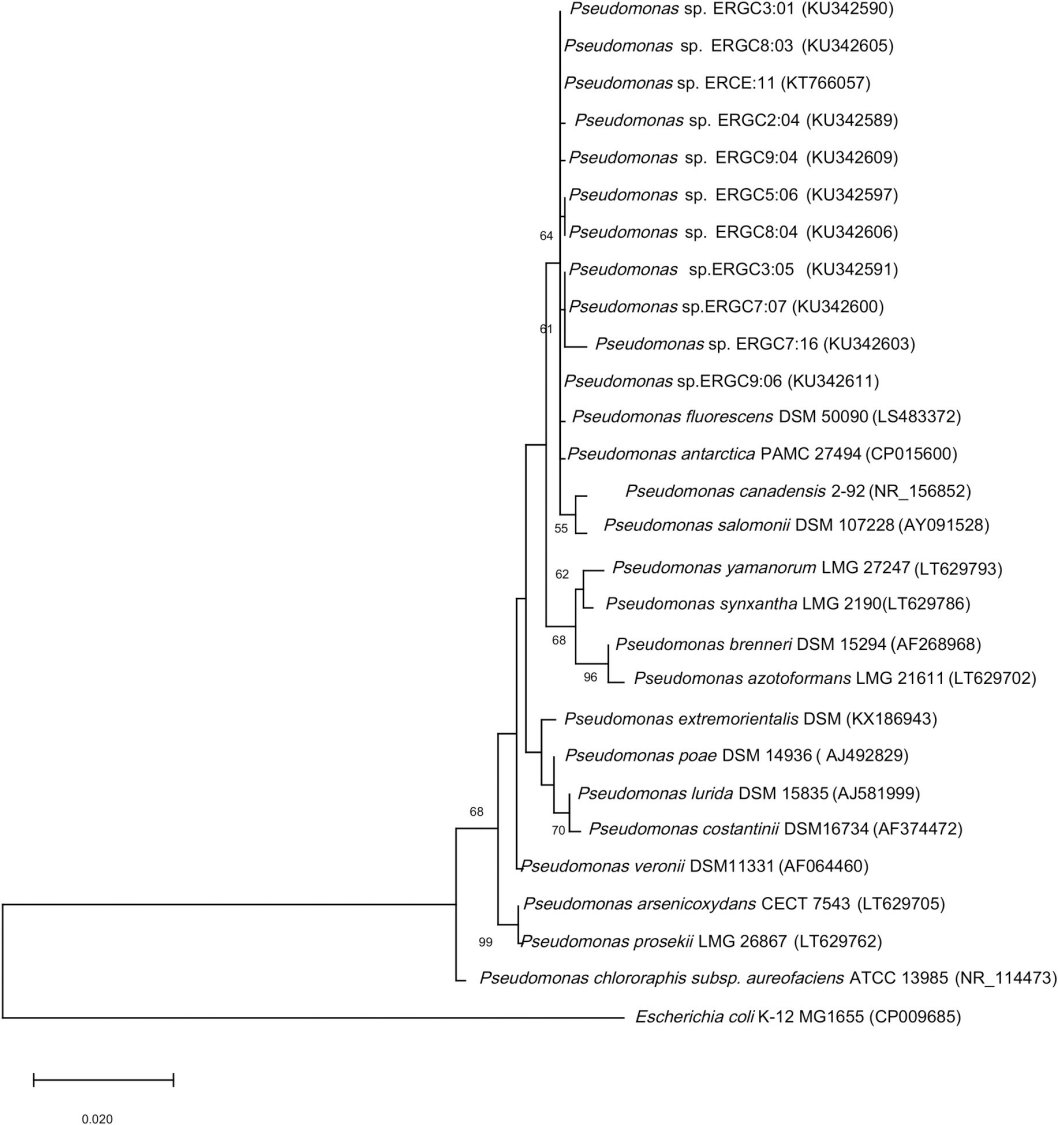
Maximum likelihood tree based on the partial 16S rRNA gene sequences of the 11 *Pseudomonas* strains and closely related validly published strains of the *Pseudomonas* genus obtained from NCBI database. The 11 *Pseudomonas* strains show clustering with both *P*. *fluorescens* and *P*. *antarctica*. *E*. *coli* K12 MG1655 was used as the outgroup organism. The tree was constructed using MEGA X software based on the Kimura 2-parameter model with 1000 bootstrap replications, and the scale bar corresponds to the average number of nucleotide substitutions per site. Accession numbers are given in parenthesis.

Molecular characterization based on the 16S rRNA gene sequence (accession numbers provided in S1 Table) showed the closest sequence similarity (top-hit) of all eleven strains with *Pseudomonas fluorescens* DSM 50090^T^. However, the sequences showed a similarity of above 99% (threshold) with other *Pseudomonas* strains. The phylogenetic tree was constructed with all the neighboring sequences showing >99% similarity to determine the affiliations of these eleven strains. However, in the phylogenetic tree, the strains were close to both *P*. *fluorescens* DSM 50090 and *P*. *antarctica* PAMC 27494 (Fig 2), which creates a taxonomic discrepancy. To clarify the phylogenetic affiliation of the strains, we partially sequenced the housekeeping genes *gyrB*, *ileS*, *nuoD*, *recA*, and *rpoD*, and concatenated the sequences to form a single sequence of 2526 bp. We analyzed the maximum likelihood trees of each housekeeping gene (S2 Fig).

**Fig 3:**
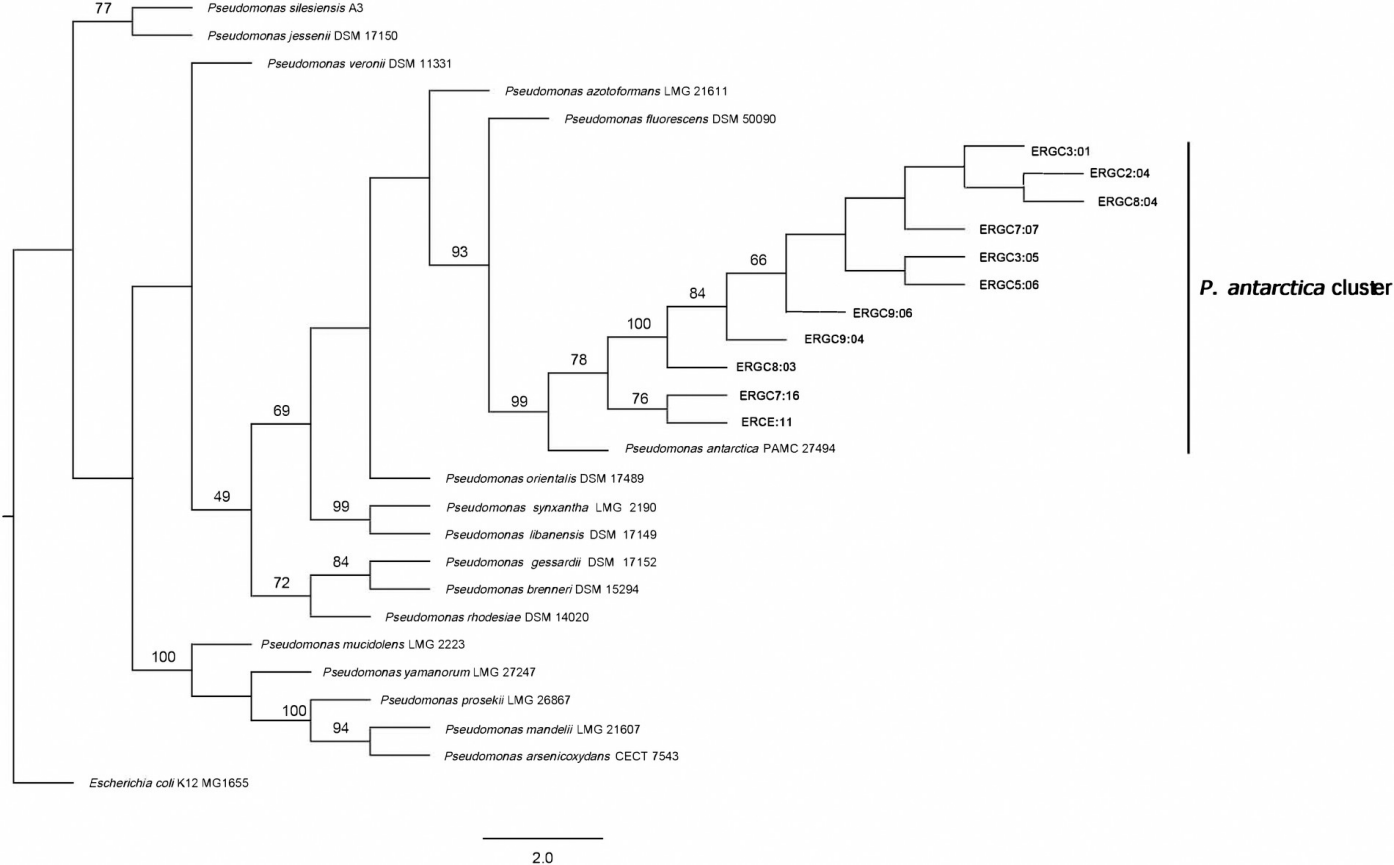
Phylogenetic tree of 11 *Pseudomonas* study strains based on the concatenated sequences of protein-coding genes *gyrB-ileS-nuoD-recA-rpoD* using RAxML. Support values are calculated from 1000 rapid bootstrap replicates (BT). BT values of 50 or more are indicated at branching points. The scale bar corresponds to the average number of nucleotide substitutions per site. Our test strains are marked in bold, that form a separate cluster with *P*. *antarctica* PAMC 27494. *E*. *coli* K12 MG1655 was used as the outgroup organism. Partitionfinder v2.1.1 was used to determine the best-fit partitioning schemes and substitution models of molecular evolution.

In the case of the *gyrB* gene, the eleven *Pseudomonas* strains were grouped in a single cluster with *P*. *antarctica* and *P*. *fluorescens* strains, of which nine of the strains showed closer relatedness to *P*. *antarctica* strain based on evolutionary distances. With *ileS*, among eleven strains, two of the strains ERCE:11 and ERGC7:16 showed the clear affiliation to *P*. *antarctica* PAMC 27494, while *P*. *fluorescens and P*. *azotoformans* were the next closest neighbors. All of the eleven strains formed a separate cluster with *P*. *antarctica* and *P*. *veronii* in the *nuoD* gene-based tree, with a higher bootstrap value in the case of *P*. *antarctica*. Similarly, with *recA*, the eleven strains formed one separate clade with *P*. *antarctica* strain, while ERCE:11 exhibited clear associations to *P*. *antarctica*. Phylogeny with *rpoD* revealed clustering of the eleven strains with *P*. *antarctica*, *P*. *fluorescens*, and *P*. *orientalis* in a single clade, where ERGC7:16, ERGC8:03, and ERCE:11 formed a distinct clustering with *P*. *antarctica*. Thus, the single-gene trees based on housekeeping gene sequences gave a higher resolution than the 16S rRNA gene tree alone. The ML tree with MLSA showed clustering of the eleven strains with *P*. *antarctica* PAMC 27494 with a bootstrap value of 99, while *P*. *fluorescens* formed a sister taxon with a lower bootstrap value (Fig 3).

The nucleotide polymorphism statistics of 11 strains for each gene and five concatenated genes are displayed in Table 1. Neutrality test, i.e., Tajima’s D, was performed to test whether any of the tested genes have undergone recent selective events in each locus. There were no significant Tajima’s D values for any housekeeping genes, indicating a neutral evolution pattern. A significant negative Tajima’s D value for the 16S rRNA gene depicts an excess of rare alleles resulting from a recent selective sweep. The *gyrB* gene showed the most parsimony informative sites with 318, followed by *nuoD* with 291, *rpoD* with 258, *recA* with 257, and *ileS* with 88. These values were higher than that of the 16S rRNA gene, hence providing more genetic information. The mean G+C content of the genes varied between 53.4 to 61.9%. The neutrality of sequence polymorphism was also checked by the ratio of non-synonymous (*d*_N_) to synonymous (*d*_S_) nucleotide substitution values. Variation in the *d*_N_/*d*_S_ ratio was observed for all five genes. The *d*_N_/*d*_S_ < 1 for the housekeeping genes *recA* and *rpoD* could be predictive that these genes were under purifying selection, while the remaining genes had values higher than 1, indicating their diversifying selection [49]. With MLSA, the concatenated sequences of 2526 bp showed a parsimony-informative site of 1140 and a G+C content of 56.1 mol%.

**Table 1:** Gene characteristics of 11 *Pseudomonas* strains.

Locus	Length (bp)	*S*	P	Mean G+C content (%)	K2P distance (range)	*π*	*Ɵ*_*W*_	Tajima’s D	*d*_N_/*d*_S_
**16S rRNA**	1326	207	33	53.4	0–0.00455	0.01709	0.04067	-2.25705	0.97691
***gyrB***	504	379	318	53.4	0–0.0144	0.23835	0.20022	0.74543	1.34062
***ileS***	548	242	88	55.3	0–0.00933	0.06923	0.11407	-1.53265	2.33873
***nuoD***	526	351	291	58.5	0–0.00389	0.13681	0.17769	-0.89988	1.33249
***recA***	444	308	257	61.9	0–0.00693	0.18444	0.18541	-0.02047	0.46937
***rpoD***	494	295	258	61.1	0–0.00208	0.17852	0.16259	0.38264	0.92327
**MLSA** *	2526	1604	1140	56.1	0–0.00695	0.15881	0.17089	-0.27819	1.07161

*S*, number of segregating sites; P, parsimony informative sites; *π*, nucleotide diversity; *Ɵ*_*W*_, theta (per site) from *Eta*; *d*N/*d*S, the ratio of non-synonymous to synonymous changes

*, *gyrB-ileS-nuoD-recA-rpoD* concatenated gene sequences.

The mean genetic distance calculated using the Kimura 2-Parameter distance model was minimum with *P*. *antarctica* PAMC 27494 (0.73%), while with *P*. *fluorescens* DSM 50090^T^ the value was higher (3.65%). The distance values further increased with other close neighbors (S4 Table). Based on the amplified DNA bands of ERIC-PCR products, clustering analysis revealed three main groups (S3 Fig). Cluster 1 contained ERGC9:04 and ERGC5:06; cluster 2 comprised two sub-clusters, one with two of the strains ERGC3:01 and ERGC3:05 and the other with six strains; while cluster 3 contained a single strain ERGC8:04.

### Adaptational characteristics

**Fig 4:**
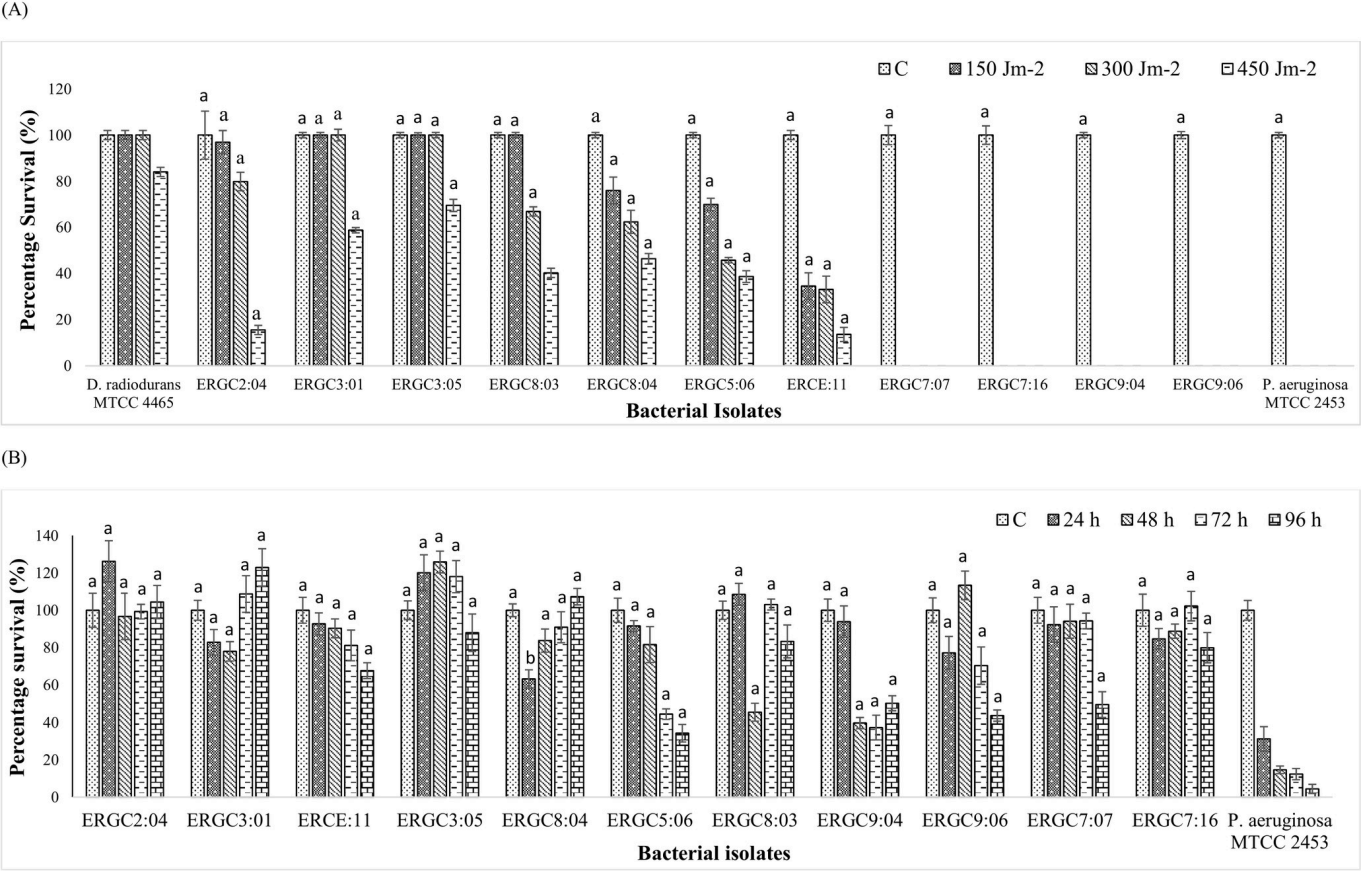
Tolerance of the 11 *Pseudomonas* strains to (A) UV-C irradiation at 150, 300, and 450 Jm^-2^, (B) freezing for 96 h, whereby percentage survival was calculated relative to the colony count of non-irradiated and unfrozen control as 100%, respectively. The mean of triplicate colony count was used to calculate percentage survival. Error bars denote the standard deviation of the mean of the three biological replicates (n = 3). Letters a, b denotes significant differences between the (A) positive control strain (*Deinococcus radiodurans* MTCC 4465) and test strains and (B) negative control strain (*Pseudomonas aeruginosa* MTCC 2453) and test strains; at p<0.0001 and p<0.0002, respectively, using one-way ANOVA by Dunnett’s multiple comparison test.

Most of the strains showed survivability >65% after preliminary screening at 150 Jm^-2^ UV-C doses. Further testing at 300 Jm^-2^ screened for the strains that could efficiently survive the given dose of UV-C radiation. Among all, ERGC3:01 and ERGC3:05 showed 100% survival on UV-C exposure of 300 Jm^-2^. ERGC2:04, ERGC8:03, and ERGC8:04 showed survivability above 60%, while ERGC5:06 and ERCE:11 showed comparatively lesser tolerance of <50% (45% and 33% respectively) to the tested UV-C dosage. On subsequent exposure to 450 Jm^-2^ UV-C dosages, two strains, i.e., ERGC3:01 and ERGC3:05, showed remarkable survival percentages of 58% and 69%, respectively. This was closely followed by ERGC8:04, ERGC8:03, and ERGC5:06. ERGC5:06 and ERCE:11 diminished to 15% and 13%, respectively. Four strains, i.e., ERGC7:07, ERGC7:16, ERGC9:04, and ERGC9:06, and the mesophilic control strain *Pseudomonas aeruginosa* MTCC 2453 showed zero survivability after all the given UV-C treatments (Fig 4A). UV-C resistance of bacteria was determined using fractionated fluences in an earlier study [50].

The colony counts of most test strains increased after subsequent freezing conditions, except ERGC5:06 and ERCE:11, which showed decreased survival than the unfrozen control during the 96 h freezing experiment. Among all, ERGC2:04 maintained its percentage survival till 96 h. ERGC3:01 showed a decreased survivability for 48 h, after which an increased survival was observed, while ERGC3:05 displayed increasing colony counts for 48 h followed by a steep decrease in survival. ERGC7:07 maintained its CFUs with slight variations till 72 h, decreasing thereafter. Some strains like ERGC7:16 and ERGC8:03 showed unusual responses by exhibiting a rise and a sharp decline in percent survival only at one point over the time course, respectively. ERGC8:04 exhibited increased survivability right after an initial decrease at 24 h of freezing. It was observed that ERGC9:04 showed decreasing survivability with a slight increase at 96 h, and ERGC9:06 also showed decreasing survival after a maximum survival at 48 h, while the survivability of ERGC5:06 and ERCE:11 decreased linearly during the entire experiment. Overall, the negative effect of freezing was most pronounced in ERGC5:06, ERGC7:07, ERGC7:16, and ERGC9:06 that reduced to <50% survival percentage by 96 h. The mesophilic control strain *P*. *aeruginosa* exhibited diminished survivability of 4% by 96 h of freezing (Fig 4B).

### Cold-active enzyme estimation

Qualitative screening for four different extracellular hydrolytic enzymes in eleven strains of *Pseudomonas* showed the presence of one or more enzymatic activity at 10°C. Two of the strains, ERGC5:06 and ERGC8:03, were positive for all of the four enzymes tested. All eleven strains predominantly exhibited lipolytic and proteolytic activities, followed by five strains with cellulolytic and four with amylolytic activity, respectively.

**Fig 5:**
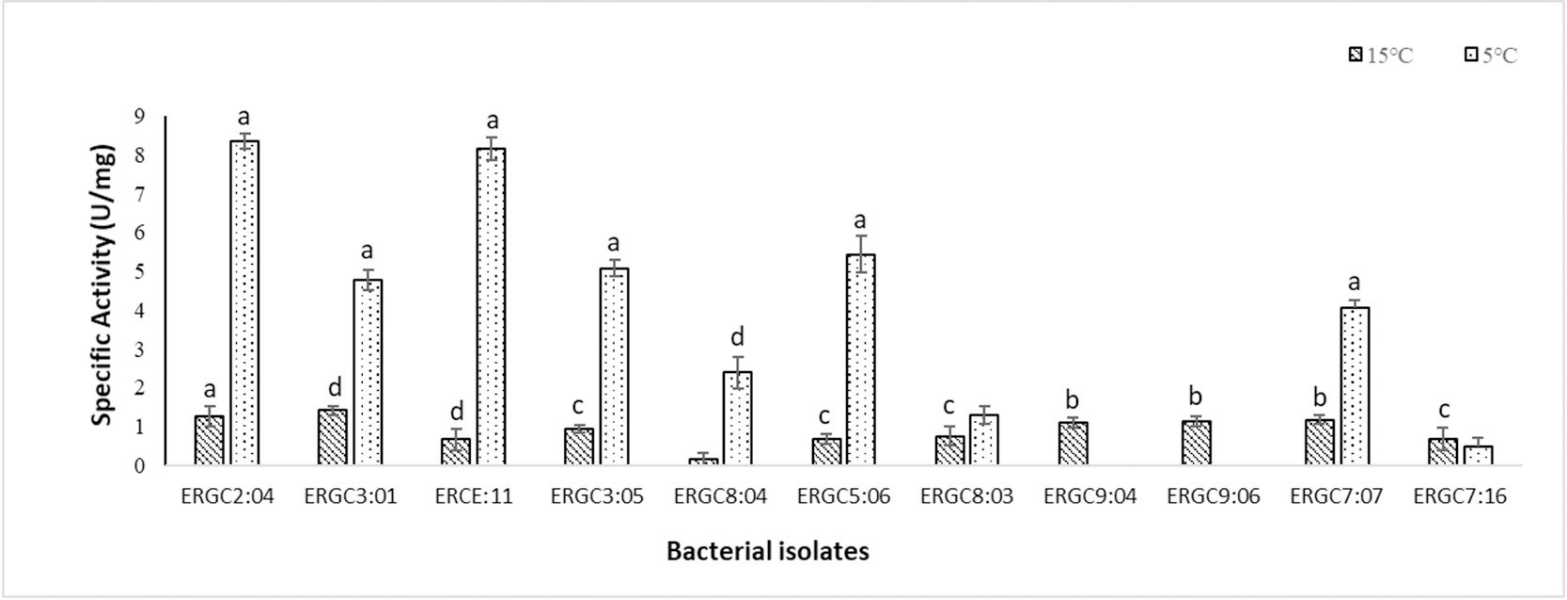
Protease activities of 11 *Pseudomonas* strains at 5°C and 15°C, with casein as substrate. Error bars denote the standard deviation of the mean of the three biological replicates (n = 3). Letters a, b, c, d denote significant differences between the reaction control and reaction test at p<0.0001, p<0.0002, p<0.0003, p<0.0004, respectively, using one-way ANOVA by Dunnett’s multiple comparison test (No letter = non-significant value).

We conducted the quantitative estimation of protease, where the extracellular enzyme of eleven bacterial strains was first harvested after 52 h of production at 20°C. The activities were assayed using casein as a substrate. Eight strains, namely ERGC2:04, ERGC3:01, ERGC3:05, ERGC5:06, ERGC7:07, ERGC8:03, ERGC8:04, and ERCE:11, showed higher specific activities at 5°C as compared to 15°C. Among all the strains, the maximum specific activity of 8.46 U/mg was exhibited by *Pseudomonas* sp. ERGC2:04 at 5°C. No activity was observed at 5°C in the case of strains ERGC9:04 and ERGC9:06, but both showed considerable activity at 15°C. ERGC3:01 displayed the highest activity of 1.36 U/mg at 15°C (Fig 5).

### Genome assembly and annotation of strains ERGC3:01 and ERGC3:05

Two representative strains, ERGC3:01 and ERGC3:05, were selected for genome sequencing as these two strains showed superior multi-adaptational characteristics of tolerance to UV radiation and freezing, as well as quantitative protease enzyme production at 5°C. BUSCO analysis of the assembled genomes was performed with the pseudomonadales_odb10 lineage dataset. The assembled genome of ERGC3:01 strain showed that 89.6% of assessed genes were identified and complete (single copy = 89.3%, duplicated = 0.3%). CheckM analysis of the same strain showed completeness of 98.43% and contamination of 0.82%. ERGC3:05 strain showed that a total of 89.3% genes were assessed using BUSCO (single copy = 89%, duplicated = 0.3%). CheckM analysis of the ERGC3:05 genome showed completeness of 98.41% and contamination of 0.91%. BUSCO and CheckM analysis results specified that the genome assemblies were good.

### Genomic features of strains ERGC3:01 and ERGC3:05

MinION sequencing resulted in two contigs of 6,494,859 bp and 39,967 bp for ERGC3:01 and a single contig of 6,496,199 bp for ERGC3:05 with mean coverages of 153X and 2015X, respectively (S4 Fig, S5 Table). No plasmids were detected in any of the strains. The PGAP predicted a total of 6,075 genes in ERGC3:05, out of which 5,985 were coding sequences, and 90 were RNA genes. Similarly, PGAP annotation resulted in 6,016 genes in ERGC3:05, including 5,926 coding sequences and 90 RNA genes.

### Phylogenomic tree and comparative genomics

**Fig 6:**
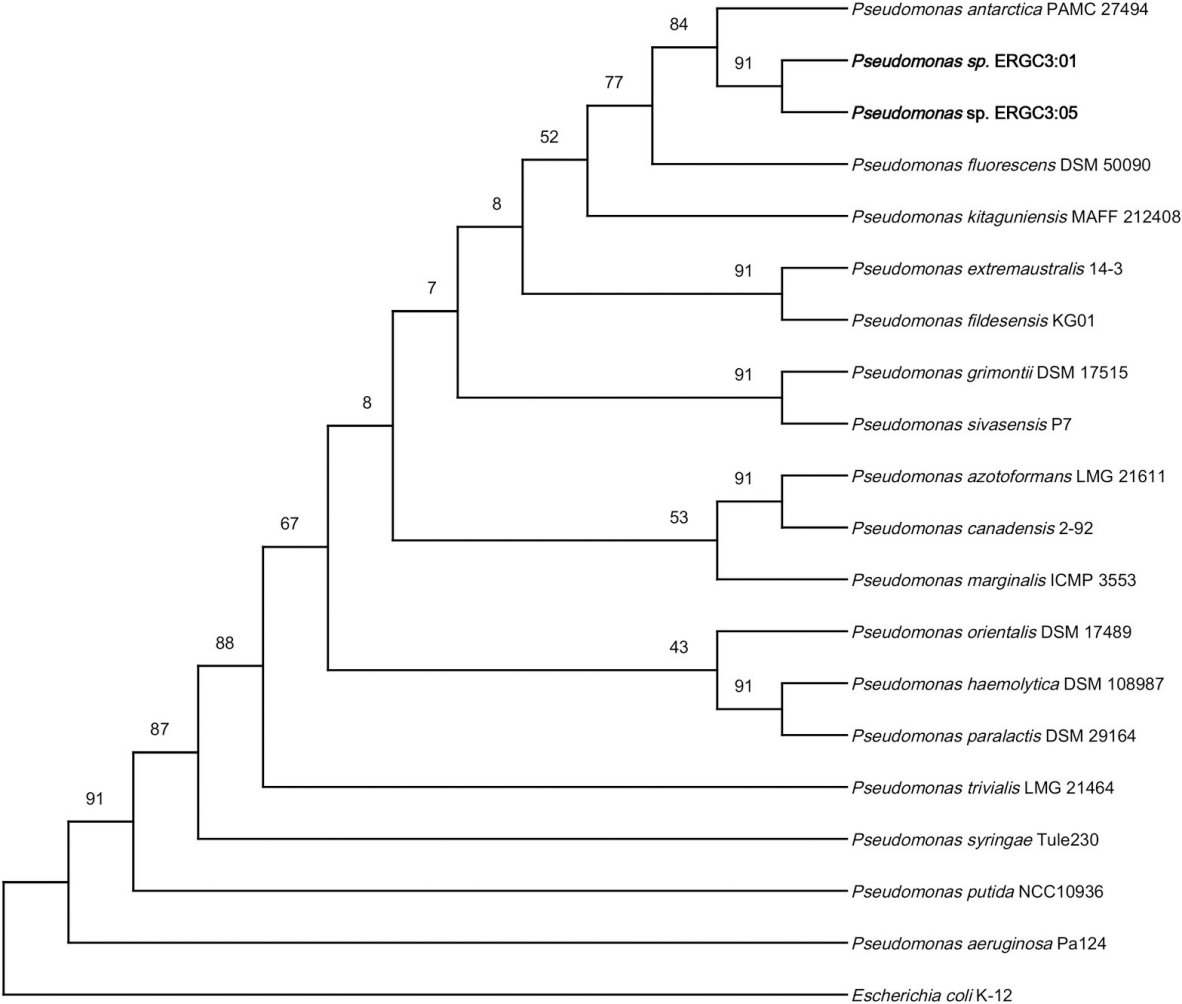
Phylogenomic tree of representative *Pseudomonas* strains based on 91 core genes using the UBCG tool. The tool extracted 91 core genes from the genomes of nearest neighbors and prepared the tree using RaxML. Support values are calculated from 1000 rapid bootstrap replicates (BT) and BT values are indicated at branching points. Our test strains are marked in bold, *E*.*coli* K12 MG1655 was used as the outgroup organism.

The phylogenetic tree constructed using bacterial core genes extracted from the whole genomes of ERGC3:01 and ERGC3:05 strains showed their close relatedness with *Pseudomonas antarctica* PAMC 27494 (Fig 6). The ANI and dDDH values of the genome pairs are given in Table 2. The strain ERGC3:01 showed the highest ANI value of 99 and the maximum dDDH value of 91.2 with *P*. *antarctica* PAMC 27494. Likewise, the ERGC3:05 strain showed the highest ANI value of 98.94 and maximum dDDH value of 91.1 with *P*. *antarctica* PAMC 27494.

**Table 2:** Average nucleotide identity (ANI) and digital DNA: DNA hybridisation (dDDH) values between genomic pairs.

S. No.	Pair	ANI	dDDH
1	ERGC3:01: *P*. *antarctica* PAMC 27494	99	91.2
2	ERGC3:05: *P*. *antarctica* PAMC 27494	98.94	91.1
3	ERGC3:01: *P*. *fluorescens* DSM 50090	94.68	58.3
4	ERGC3:05: *P*. *fluorescens* DSM 50090	94.67	58.2
5	ERGC3:01: ERGC3:05	99	99

### Genomic insights into adaptational strategies

The supraglacial strains were able to survive the freezing and UV-C exposures; hence, to assess the molecular mechanisms behind their adaptation, genome mining was conducted for the known cold and radiation-associated genes in two potential strains ERGC3:01 and ERGC3:05. Both the strains carried important genes encoding cold-active chaperones, proteins for osmotic and oxidative stress response, DNA repair, membrane/cell wall modifications, and general stress response (Table 3). Cold shock protein and molecular chaperones were observed in multiple copies in both the genomes. The bacterial genomes also carried three copies of universal stress protein that are produced against environmental stress factors. Proteins involved in cell membrane modification that includes fatty acid desaturase, sterol desaturase, and 3-oxoacyl-(acyl-carrier-protein) reductase occurred in multi-copies in the genomes. Besides, the genomes harboured many copies of glycine betaine and choline ABC transporter genes that enable the uptake of compatible solutes during osmotic stress. As a defence mechanism against DNA damage caused by UV exposure, the bacterial genomes showed numerous genes that participate in DNA repair. These include cryptochrome/photolyase family protein; DNA glycosylases; DNA repair proteins RadC, RadA, RecN, RecO, RecF; recombinase RecA; DNA helicase RecQ, RecG, UvrD; DEAD box helicases; mismatch repair proteins MutL, MutM, MutS, and many others. Oxidative stress response proteins were present in the genomes, including catalase, superoxide dismutase, peroxiredoxin, peroxidase, and thioredoxins.

**Table 3:** Genes encoding known cold & stress response and DNA repair proteins with accession number/locus tag as predicted in the genomes of *Pseudomonas* sp. ERGC3:01 and ERGC3:05.

Category and Gene name	ERGC3:01	ERGC3:05
**Cold active chaperones**		
cold shock domain-containing protein cspD	MBX7277211	QZC92650
cold shock domain-containing protein	MBX7277726	QZC96765
cold-shock protein	MBX7278198, MBX7278220, MBX7277725, MBX7277502, MBX7275531, MBX7275436	QZC96311, QZC96331, QZC96766, QZC96971, QZC94246, QZC94333
ATP-dependent chaperone ClpB	K2E95_04060	
copper chaperone PCu(A)C	MBX7276217, K2E95_02930	QZC94865, QZC93568
RNA chaperone Hfq	MBX7274801	QZC94925
protein-export chaperone SecB	MBX7274654	QZC95060
Hsp33 family molecular chaperone HslO	MBX7274551	QZC95155
zinc metallochaperone GTPase ZigA	MBX7279164	QZC95413
fimbria/pilus periplasmic chaperone	MBX7279013, MBX7278561, MBX7276807, MBX7276730, MBX7276706	QZC93117, QZC94453, QZC94521, QZC95560, QZC95985
radical SAM family heme chaperone HemW	K2E95_27730	K2E96_05700
pyrroloquinoline quinone biosynthesis peptide chaperone PqqD	MBX7278758	K2E96_06545
molecular chaperone DnaK	MBX7278493	QZC96056
molecular chaperone DnaJ	MBX7278492	QZC96057
molecular chaperone HtpG	MBX7275935	QZC93856
molecular chaperone	MBX7276723, MBX7276806, MBX7277317, MBX7277436, MBX7277440	QZC92558, QZC93025, QZC93098, QZC97027, QZC97030
co-chaperone HscB	K2E95_24260	K2E96_09190
Fe-S protein assembly chaperone HscA	MBX7278341	QZC96200
co-chaperone GroES	MBX7278279	QZC96258
chaperonin GroEL	MBX7278278	QZC96259
CcoQ/FixQ family Cbb3-type cytochrome c oxidase assembly chaperone	MBX7277848	QZC96649
outer membrane lipoprotein chaperone LolA	MBX7277205	QZC92657
**Membrane/cell wall alteration**		
fatty acid desaturase	MBX7274502, MBX7274512, MBX7278138	QZC95190, QZC96375, K2E96_02620
sterol desaturase family protein	MBX7279018, MBX7277397	QZC95555, QZC97065
3-oxoacyl-ACP reductase	MBX7274904, K2E95_12510	QZC94823, QZC97271
3-oxoacyl-ACP reductase FabG	MBX7274719, MBX7277921, MBX7278025	QZC95003, QZC96485, QZC96578
UDP-N-acetylglucosamine 1-carboxyvinyltransferase	MBX7275138	K2E96_28800
acyl-ACP—UDP-N-acetylglucosamine O-acyltransferase	MBX7275514	QZC94263
**Osmotic Stress**		
glycine betaine/L-proline ABC transporter ATP-binding protein	MBX7274672	QZC95044
proline/glycine betaine ABC transporter permease	MBX7274671	K2E96_01575
glycine-betaine demethylase subunit GbcA	MBX7278801	K2E96_06290
glycine-betaine demethylase subunit GbcB	MBX7278800	QZC95762
glycine betaine ABC transporter substrate-binding protein	MBX7277626, MBX7275918, MBX7275069, K2E95_18825	QZC96857, QZC93873, QZC94675, K2E96_14605
glycine betaine/L-proline transporter ProP	MBX7276929	QZC92916
glycine betaine/L-proline ABC transporter ATP-binding protein	MBX7274672	QZC95044
choline ABC transporter substrate-binding protein	MBX7274677, MBX7278811, MBX7278818, MBX7278819	QZC95325, QZC95743, QZC95751, QZC97102
choline ABC transporter ATP-binding protein	K2E95_27255	QZC95741
choline ABC transporter permease subunit	MBX7278819	QZC95742
choline dehydrogenase	MBX7278823	QZC95738
choline transporter BetT	MBX7274538	QZC97113
sodium/proline symporter PutP	MBX7274748	QZC94976
proline dehydrogenase	MBX7278851	QZC95711
sarcosine oxidase subunit beta	K2E95_27120	QZC95766
sarcosine oxidase subunit delta	MBX7278796	QZC95767
sarcosine oxidase subunit alpha	MBX7278795	K2E96_06325
sarcosine oxidase subunit gamma family protein	MBX7278794	QZC95768
osmoprotectant NAGGN system M42 family peptidase	MBX7277643	QZC96845
**Oxidative stress**		
catalase HPII	MBX7279301	QZC95288
catalase	MBX7278666	QZC95887
catalase KatB	MBX7278526	QZC96025
catalase family peroxidase	K2E95_17990	K2E96_15440
catalase family protein	MBX7275986	QZC93806
superoxide dismutase [Fe], SodB	K2E95_23175	K2E96_10275
superoxide dismutase	MBX7275121	QZC94629
peroxiredoxin	MBX7275659, MBX7276813, MBX7278970, K2E95_05920	QZC95596, QZC93019, QZC94122, K2E96_27505
alkyl hydroperoxide reductase subunit F	MBX7276812	QZC93020
organic hydroperoxide resistance protein	MBX7275854	QZC93936
thioredoxin TrxC	MBX7274738	QZC94985
thioredoxin TrxA	MBX7279006	QZC95567
thioredoxin	MBX7275845, MBX7278645	QZC95905, QZC93945
thioredoxin-disulfide reductase	MBX7278404	QZC96141
thioredoxin fold domain-containing protein	MBX7278295	QZC96245
glutathione peroxidase	MBX7278407, MBX7275868	QZC96138, K2E96_24460,
thiol peroxidase	MBX7276452	QZC93347
deferrochelatase/peroxidase EfeB	MBX7276550	K2E96_20090
Dyp-type peroxidase	MBX7277256	K2E96_15740
catalase family peroxidase	K2E95_17990	K2E96_15440
**DNA repair**		
cryptochrome/photolyase family protein	MBX7277059	QZC92801
bifunctional DNA-formamidopyrimidine glycosylase/DNA-(apurinic or apyrimidinic site) lyase	MBX7278913	QZC95650
uracil-DNA glycosylase	MBX7275643	QZC94139
G/U mismatch-specific DNA glycosylase	MBX7277100	K2E96_16765
A/G-specific adenine glycosylase	MBX7274640	QZC95074
DNA-3-methyladenine glycosylase	MBX7278602	QZC95337
DNA-3-methyladenine glycosylase I	MBX7276985, MBX7277177, MBX7279253	QZC92686, QZC92869, QZC95337
DNA-3-methyladenine glycosylase 2 family protein	K2E95_23310	QZC96341
DNA repair protein RadC	MBX7279073	QZC95498
DNA repair protein RadA	MBX7278524	QZC96027
DNA repair protein RecO	MBX7275285	QZC97315
DNA repair protein RecN	MBX7278495	QZC96054
recombinase RecA	MBX7275382	QZC94384
recombination regulator RecX	MBX7275383	QZC94383
recombination mediator RecR	MBX7277857	QZC96639
single-stranded-DNA-specific exonuclease RecJ	MBX7278289	QZC96252
DNA helicase RecQ	K2E95_20750	QZC96729
DNA replication/repair protein RecF	K2E95_29790	K2E96_03615
ATP-dependent DNA helicase RecG	MBX7279096	QZC95477
exodeoxyribonuclease V subunit beta RecB	K2E95_24920	K2E96_08525
exodeoxyribonuclease V subunit alpha RecD	K2E95_24915	K2E96_08530
exodeoxyribonuclease V subunit gamma RecC	K2E95_24925	K2E96_08520
DNA recombination protein RmuC	K2E95_20770	K2E96_12655
recombination-associated protein RdgC	MBX7275978	QZC93812
replication-associated recombination protein A	MBX7277204	QZC92658
DNA helicase II uvrD/ mutU/ recL	K2E95_29260	K2E96_04155
UvrD-helicase domain-containing protein	K2E95_13555	QZC93235
DDE-type integrase/transposase/recombinase	K2E95_21775	K2E96_11665
DNA mismatch repair endonuclease MutL	K2E95_02535	K2E96_00810
DNA mismatch repair protein MutS	MBX7275360	QZC94404
bifunctional DNA-formamidopyrimidine glycosylase/DNA-(apurinic or apyrimidinic site) lyase mutM	MBX7278913	QZC95650
A/G-specific adenine glycosylase mutY	MBX7274640	QZC95074
DNA/RNA non-specific endonuclease	MBX7276127, MBX7277616	QZC95220, QZC96863
endonuclease III	MBX7275355	QZC94408
DEAD/DEAH box helicase	MBX7275863, MBX7277191, MBX7278548, MBX7278853, K2E95_08185, K2E95_21040, K2E95_26210	QZC92672, QZC93928, QZC95998, QZC95708, K2E96_25235, K2E96_12385, K2E96_07230
**General Stress response**		
GlsB/YeaQ/YmgE family stress response membrane protein	MBX7279137	QZC95440
universal stress protein	MBX7276970, MBX7275848, MBX7275726	QZC92880, QZC93942, QZC94059
general stress protein	MBX7276221	K2E96_22140
peroxide stress protein YaaA	MBX7275212	QZC94541
envelope stress response protein PspG	MBX7275042	QZC94697
50S ribosomal protein L25/general stress protein Ctc	MBX7275001	QZC94734

## Discussion

The presence of bacterial communities in supraglacial habitats has been documented before, and many reports are available on the occurrence of *Pseudomonas* in the supraglacial ecosystems [51–55]. It has been suggested that microbes in supraglacial environments are involved in carbon and nutrient cycling [5]. Being a common inhabitant of a cold environment, *Pseudomonas* is considered to be of scientific and industrial significance as it is a metabolically versatile organism and an excellent producer of extracellular enzymes [56].

Our present study focussed on exploring, for the first time, the bacterial diversity from a supraglacial ecosystem of the East Rathong glacier in Sikkim Himalaya with possible bioprospection and adaptational studies. In the course, many unique morphotypes of bacteria were obtained by culturing, and 32 of them were identified up to species level. Among 32 identified bacteria, 21 of them belonged to *Pseudomonas*, of which 11 strains notably showed the closest similarity to *P*. *fluorescens*.

In a previous study, MLSA has proven to be a useful tool for identification and species discrimination of *Pseudomonas* sp. [57]. The genes that are regularly used in the taxonomy of *Pseudomonas* include those involved in replication and translation [58]. The genes *rpoD* and *gyrB* are employed broadly in new species descriptions within the genus. Mulet et al. [59] reported that the sequence analysis of four housekeeping genes viz. *16S rRNA*, *gyrB*, *rpoB*, and *rpoD* helped clarify the phylogeny of *Pseudomonas* strains. In one study, compared to the use of three or four genes for characterization of *Pseudomonas* strains, concatenation of six genes viz. *16SrRNA*, *aroE*, *gyrS*, *glnS*, *ileS* and *rpoD*, led to stronger analysis of the Antarctic strains [9]. The differentiation at strain level was high and robust on applying this set of housekeeping genes i.e. *glnS*, *gyrB*, *ileS*, *nuoD*, *recA*, *rpoB*, and *rpoD* as reported by Andreani et al. [29]. Based on the phylogenetic analysis of concatenated sequences of five loci i.e., *gyrB*, *ileS*, *nuoD*, *recA*, and *rpoD*, which has been more reliable than the traditional 16S rRNA gene, we inferred that our strains could be assigned to *P*. *antarctica*, based on the branching and bootstrap values. Although they show a very close association to *P*. *fluorescens*, they formed a distinct cluster with *P*. *antarctica*, which we are referring to as *P*. *antarctica* cluster. Individual phylogenies of the genes were also analyzed, which provided a clearer picture. Especially *recA* gene tree placed all the strains in one cluster with *P*. *antarctica*, while other single genes did not reveal clear demarcation among species. The assigning of the 11 strains to *P*. *antarctica* was also supported by the genetic distance calculated by the Kimura-2-parameter model, which showed closer relatedness of these strains to *P*. *antarctica* (0.00734) than with *P*. *fluorescens* (0.0365). To estimate the genetic distance, evolutionary distances between two sequences are calculated based on the number of nucleotide base substitutions [33]. The genome relatedness indices further validated the results of MLSA and K2P genetic distance calculation. The ANI and dDDH values of the two representative strains ERGC3:01 and ERGC3:05 with *P*. *antarctica* are way higher than the threshold values of 95 and 70, respectively, which suggests that the strains belong to the species *P*. *antarctica*. The type strain *P*. *fluorescens* NCTC 10038 is a mesophilic strain with an optimal growth temperature of 30°C (NCBI database), while our strains are all psychrotrophic with optimal growth temperature of 20°C and probably shows more relatedness to the Antarctic strain *P*. *antarctica* PAMC 27494.

Highly similar 16S rRNA gene sequences between individuals do not essentially correspond to the high similarity of the genomes of different strains [60]. In our case, although the strains showed very similar 16S rDNA patterns, they differed by their morphological and physiological characteristics. Repetitive element-based PCR (Rep-PCR) is a reliable and widely used method to differentiate between the closely related strains of bacteria. Among Rep-PCR, Enterobacterial Repetitive Intergenic Consensus (ERIC) elements are commonly used for the molecular typing of Gram-negative bacterial genera [61]. The patterns of ERIC-PCR suggested genetic heterogeneity among the investigated strains.

The bacterial strains that we obtained from the glacial surface are psychrotrophic and are not strict psychrophiles, as they grew best at 20°C rather than 4°C, and the maximum limit of growth was 28°C for most and 37°C for a few. Frequent exposure of bacterial cells in glacier ice to repeated freeze-thawing cycles and high ultraviolet radiations is common, and microorganisms isolated from such environments are expected to resist multiple harsh conditions. They do so by developing multiple cold-associated adaptational strategies, including expression of cold-active chaperones and cold-active enzymes, cell membrane or cell wall modifications, activation of osmotic and oxidative stress responses, upregulation of DNA repair enzymes, and other stress response proteins [11, 62].

Physiological assays were performed to evaluate the characteristics expected from bacteria isolated from a unique cold niche. Since UV-C radiation is the most effective bactericidal agent, this provides an easy means for assessing highly radiation-resistant microorganisms [63, 64]. Bacteria isolated from high-altitude glacier ecosystems are likely to be UV-resistant owing to their constant exposure to UV radiations with an increased reflection by ice [65]. Seven strains were more tolerant than the mesophilic *Pseudomonas aeruginosa* MTCC 2453 in the tested UV-C doses. Two of the strains, ERGC3:01 and ERGC3:05, efficiently survived the dosage of 300 Jm^-2^, equivalent to *Deinococcus radiodurans* MTCC 4465 (*p* < 0.0001). In fact, the given strains showed efficient survivability even at 450 Jm^-2^. Five strains could tolerate the UV-C exposures with decreased survival percentage, while four could not survive any of the tested dosages. This depicts a variation in traits even among the same bacterial species obtained from the same niche. DNA repair mechanisms, including mismatch repair, excision repair, recombination repair, photoreactivation, etc. have been suggested to be the common strategies for surviving the DNA damage caused by high UV radiations [63].

While UV-B has been associated with both direct and indirect damage *viz*. oxidative stress and protein denaturation, UV-C chiefly causes direct effect by forming cyclobutane pyrimidine dimers and photoproducts [65]. DNA repair genes are crucial for survival from radiation-induced DNA damage. The genomes of glacier isolates *Pseudomonas* sp. ERGC3:01 and ERGC3:05 possess many genes that encode DNA repair enzymes. Cryptochrome/photolyase family protein was observed in the ERGC3:05 genome, which includes photolyases involved in the photoreactivation process for repairing direct DNA damage [66]. Besides, genes encoding DNA glycosylases that carry out base excision repair, mismatch repair proteins such as MutL, MutS, MutU, as well as other DNA repair proteins and recombinases were observed to be carried by the bacterial isolates. The bacterial genome particularly showed a high copy number of DEAD/DEAH box helicases, which play a critical role in RNA stabilization at low temperatures [62]. Low-temperature conditions and high UV exposure conditions are often accompanied by reactive oxygen species (ROS) and toxic photochemical product formation that lead to oxidative stress [12]. The *Pseudomonas* strains seem to be well-equipped with enzymatic machinery against oxidative stress, which comprises many key antioxidant enzymes like catalase, superoxide dismutase, glutathione peroxidase, peroxidases, etc., whereby peroxiredoxin, in particular, was present in multi-copies in the ERGC3:05 genome. These antioxidant enzymes directly compensate for the detrimental effects of ROS [67].

All strains exhibited notable tolerance to freezing conditions until the entire experiment period of 96 h, which is likely as freeze-thaw is a regular process in a high-altitude environment. The tolerance of eleven glacier strains to freezing was significantly higher than the mesophilic *P*. *aeruginosa* (*p* < 0.05). Such a physiological feature probably is important to support the survival of the *Pseudomonas* strains in the glacier environment where freeze-thaw is common physiological stress. It is known that the susceptibility of bacterial cells to freeze-thaw varies with strains, their physiological state, growth conditions, and other factors [68]. Each bacterial strain within the group demonstrated a unique response to the continuous freezing and thawing treatment. Certain strains displayed a subsequent increase in colony count after different days of continuous freezing and thawing. Such an enhanced survival after freezing is common and has been documented in an earlier freeze-thaw study of ice core bacteria [69]. Microorganisms in frozen biomes are reported to be metabolically active even under frozen conditions, through specific adaptive mechanisms. Upregulation of cell wall and membrane maintenance genes, cold-shock proteins, particularly RNA helicase protein CsdA as a key protein, and alterations in cellular energy metabolism have been proposed to be relevant for survival in icy conditions [70, 71].

One noteworthy bacterial adaptation in response to the cold temperature is the production of certain DNA/RNA-binding proteins known as cold-shock proteins (CSPs), cold-active chaperones and helicases, that favour stabilization of DNA/RNA processes [62]. The genomes of both *Pseudomonas* sp. ERGC3:01 and ERGC3:05 are found to harbor CSPs in multiple copies. Molecular chaperones play an important role in coping with cold by facilitating the proper folding of proteins during cold stress [72]. Many copies of molecular chaperones and ATP-dependent chaperone ClpB were present in the genomes. Maintaining membrane fluidity is crucial for cell viability at low temperatures [11]. Increased synthesis of polyunsaturated fatty acids (PUFAs) conserves the fluidity, consequently, many genes encoding fatty acid biosynthesis proteins, including fatty acid desaturase, sterol desaturase, and 3-oxoacyl-ACP synthase, occur in multiple copies in the ERGC3:05 genome. The genetic evidence supports the observed physiological traits of these glacier bacteria, and similar genomic traits have been observed in other psychrotrophic bacteria [12, 73]. The freezing conditions of the glacier often reduce the external water activity, which leads to disruption of internal osmotic balance in bacteria [12]. Bacterial cells accumulate compatible solutes, chiefly glycine betaine, choline, and proline, to counter the osmotic stress. High-affinity transport systems like glycine betaine/choline ABC transporter systems were observed in high copy numbers in the ERGC3:05 genome. These transporters enable the uptake of nutrients and ions across biological membranes by coupling with ATP hydrolysis [74]. Besides, L-proline transporters were also carried by the bacteria, along with other related genes such as sarcosine oxidase and choline/proline dehydrogenase, and an osmoprotectant NAGGN system M42 family peptidase.

The structural and molecular adaptations in enzymes for maintaining a low-temperature catalytic activity are vital for the survival and functioning of an organism in extreme cold niches [75]. All of the strains showed some or other hydrolytic enzyme activities at low temperatures. This particular trait shows the ability of these organisms to utilize complex organic matters present in a limiting environment as a source of their nutrients. As indicated by clear halos around the colonies, besides 10°C, the production of enzymes was observed at both 5°C and 20°C. However, the colony growth and zone of clearance developed much slower in the case of incubation at 5°C. Among hydrolytic enzymes, cold-active proteases catalyze the hydrolysis of peptide bonds in proteins and peptides and constitute an important class of enzymes with huge demand in the enzyme industries [6]. In our findings, most of the strains showed better enzymatic activity at 5°C, which thus demonstrates that our strains exhibit the characteristics of cold-active enzymes. Owing to their activities at lower temperatures, these proteases may find potential industrial applications with economic and ecological benefits.

In summary, the approach of MLSA with five housekeeping genes was applied to establish the taxonomy of 11 strains of *Pseudomonas* isolated from the supraglacial site of Sikkim Himalaya. 16S rRNA gene analysis helped identify the strains at the genus level but was not significant in species identification. The MLSA results supported by mean genetic distance values and genome indices identified the strains as *P*. *antarctica*. Studies on adaptation demonstrated some of the strains, i.e., ERGC2:04, ERGC3:01, ERGC3:05, and ERCE:11, to be the finest producers of protease enzyme at 5°C, among others. Interestingly, the strains ERGC3:01 and ERGC3:05 exhibited superior tolerance to UV radiation up to 450 Jm^-2^ and comparatively good survival on 96 h of freezing, suggesting the polyadaptational attributes of these particular strains that could be exploited for bioprospection potential in varied industries like detergents, cosmetics, and pharmaceuticals.

## Supporting information

S1 Table Identification of the studied strains based on percentage similarity of the 16S rRNA genes with validly published strains and their phenotypic characterization. (PDF)

S2 Table Primers used for PCR amplification and sequencing of housekeeping genes (Andreani *et al*. 2014). (PDF)

S3 Table Specific site of sampling location (triplicate) and physicochemical properties of the collected samples. (PDF)

S4 Table Average genetic distance of the studied strains (Group 1) with nearest phylogenetic neighbours. The values are represented in percentage and calculated using Kimura 2-parameter (K2P) distance model implemented in MEGA X software.
(PDF)

S5 Table Genome features of *Pseudomonas* strains. (PDF)

S1 Fig *Pseudomonas* strains in King’s B media showing fluorescence under UV light. (PDF)

S2 Fig Phylogenetic trees of 11 *Pseudomonas* strains based on individual protein-coding genes *gyrB*, *ileS*, *nuoD*, *recA*, and *rpoD* using RAxML. Sequences of the neighbouring strains were retrieved from the complete genome sequences from GenBank database. Support values are calculated from 500 rapid bootstrap replicates. BT values of 50 or more are indicated at branching points. The scale bar corresponds to the average number of nucleotide substitutions per site. Our test strains are marked in bold. *E*. *coli* K12 MG1655 was used as the outgroup organism. Partitionfinder v2.1.1 was used to determine the best-fit partitioning schemes and substitution models of molecular evolution.
(PDF)

S3 Fig Dendrogram derived from ERIC-PCR patterns of bacterial strains based on the Jaccard similarity coefficient and UPGMA. (PDF)

S4 Fig Circular genome representations of *Pseudomonas* sp. (A) ERGC3:01 and, (B) ERGC3:05, created using CGView v2.0.2.
(PDF)
