# Supplementary material for: Multilocus sequence based identification and adaptational strategies of Pseudomonas sp. from the supraglacial site of Sikkim Himalaya
Source: PLoS One. 2022 Jan 24;17(1):e0261178. doi: 10.1371/journal.pone.0261178 (PMC8786180; doi:10.1371/journal.pone.0261178)
Supplement: S1 Table — (PDF) [file pone.0261178.s001.pdf]

Multilocus sequence based identification and adaptational strategies of *Pseudomonas* sp. from supraglacial site of Sikkim Himalaya

Srijana Mukhia, Anil Kumar, Poonam Kumari, Rakshak Kumar, Sanjay Kumar

Tables

Supplementary Table S1. Identification of the studied strains based on percentage similarity of the 16S rRNA genes with validly published strains and their phenotypic characterization.

Sl. No.	Isolates	Closest match	Accession number	% sim[a]	No. of nucleotides	Temperature range	pH range	NaCl range
1	ERCE:11	*Pseudomonas fluorescens* DSM 50090^T	KT766057	99.93	1398	4-28	4-12	1-3
2	ERGC2.04	*Pseudomonas fluorescens* DSM 50090^T	KU342589	99.86	1385	4-37	4-12	1-4
3	ERGC3:01	*Pseudomonas fluorescens* DSM 50090^T	KU342590	99.93	1396	4-37	4-12	1-4
4	ERGC3:05	*Pseudomonas fluorescens* DSM 50090^T	KU342591	99.85	1365	4-37	4-12	1-4
5	ERGC5.06	*Pseudomonas fluorescens* DSM 50090^T	KU342597	99.86	1380	4-28	4-12	1-4
6	ERGC7.07	*Pseudomonas fluorescens* DSM 50090^T	KU342600	99.86	1384	4-28	4-12	1-4
7	ERGC7:16	*Pseudomonas fluorescens* DSM 50090^T	KU342603	99.56	1355	4-28	4-12	1-4
8	ERGC8:03	*Pseudomonas fluorescens* DSM 50090^T	KU342605	99.93	1376	4-37	5-12	1-4
9	ERGC8:04	*Pseudomonas fluorescens* DSM 50090^T	KU342606	99.86	1396	4-28	4-12	1-4
10	ERGC9:04	*Pseudomonas fluorescens* DSM 50090^T	KU342609	99.85	1379	4-28	4-12	1-4
11	ERGC9:06	*Pseudomonas fluorescens* DSM 50090^T	KU342611	99.93	1356	4-37	4-12	1-5

[a]Similarity percentage with recognised type strains of validly published prokaryotic names
