# Supplementary material for: Multilocus sequence based identification and adaptational strategies of Pseudomonas sp. from the supraglacial site of Sikkim Himalaya
Source: PLoS One. 2022 Jan 24;17(1):e0261178. doi: 10.1371/journal.pone.0261178 (PMC8786180; doi:10.1371/journal.pone.0261178)
Supplement: S2 Table — (PDF) [file pone.0261178.s002.pdf]

Supplementary Table S2. Primers used for PCR amplification and sequencing of housekeeping genes (Andreani *et al*., 2014).

Primer	Sequence (5’-3’)	Gene product	Amplicon size (bp)
**gyrB_F**	GGTGGTCGATAACTCCATCG	DNA gyrase subunit B	489
**gyrB_R**	CGCTGAGGAATGTTGTTGGT		
**ileS_F**	TTCCCAATGAARGCCGGCCTGCC	Isoleucyl-tRNA synthetase	539
**ileS_R**	GGGGTGGTGGTCCAGATCACG		
**nuoD_F**	GAAGTCCTGACCTTCCTGC	NADH dehydrogenase subunit D	516
**nuoD_R**	GAAGAACTCGGCCATCATG		
**recA_F**	TGGCTGCGGCCCTGGGTCAGATC	Recombinase A	435
**recA_R**	ACCAGGCAGTTGGCGTTCTTGAT		
**rpoD_F**	CTGATCCAGGAAGGCAACATCGG	RNA polymerase sigma factor	480
**rpoD_R**	ACTCGTCGAGGAAGGAGCG		
