# Supplementary material for: Multilocus sequence based identification and adaptational strategies of Pseudomonas sp. from the supraglacial site of Sikkim Himalaya
Source: PLoS One. 2022 Jan 24;17(1):e0261178. doi: 10.1371/journal.pone.0261178 (PMC8786180; doi:10.1371/journal.pone.0261178)
Supplement: S3 Table — (PDF) [file pone.0261178.s003.pdf]

Supplementary Table S3**.** Specific site of sampling location (triplicate) and physicochemical properties of the collected samples.

Collection site	Site coordinates	Samples	pH	Temperature	Identified strains
East Rathong glacier ice core	N 27°33´.149<br>E 88°07´.406;<br>Elevation 4718m	1) Ice meltwater	6	8°C	ERGC2:04<br>ERGC3:01<br>ERGC3:05
		2) Ice meltwater	6.4	8°C	ERGC5.06<br>ERGC7.07<br>ERGC7:16<br>ERGC8:03<br>ERGC8:04
		3) Ice meltwater	6.7	8°C	ERGC9:04<br>ERGC9:06<br>ERCE:11
