# Supplementary material for: Multilocus sequence based identification and adaptational strategies of Pseudomonas sp. from the supraglacial site of Sikkim Himalaya
Source: PLoS One. 2022 Jan 24;17(1):e0261178. doi: 10.1371/journal.pone.0261178 (PMC8786180; doi:10.1371/journal.pone.0261178)
Supplement: S4 Table — The values are represented in percentage and calculated using Kimura 2-parameter (K2P) distance model implemented in MEGA X software. (PDF) [file pone.0261178.s004.pdf]

Supplementary Table S4. Average genetic distance of the studied strains (Group 1) with nearest phylogenetic neighbours. The values are represented in percentage and calculated using Kimura 2-parameter (K2P) distance model implemented in MEGA X software.

**Group 1**	**Group 2**	**Mean distance (%)**
East Rathong Glacier *Pseudomonas* strains	*Pseudomonas antarctica* PAMC 27494	0.73
	Pseudomonas fluorescens DSM 50090	3.65
	Pseudomonas azotoformans LMG 21611	5.00
	Pseudomonas libanensis DSM 17149	5.95
	Pseudomonas orientalis DSM 17489	6.01
	Pseudomonas veronii DSM 11331	6.09
	Pseudomonas gessardii DSM 17152	6.57
	Pseudomonas brenneri DSM 15294	6.6
	Pseudomonas rhodesiae DSM 14020	7.1
	Pseudomonas silesiensis A3	8.91
	Pseudomonas jessenii DSM 17150	9.1
	Pseudomonas synxantha LMG 2190	28.4
	Pseudomonas yamanorum LMG 27247	31
	Pseudomonas prosekii LMG 26867	47.3
	Pseudomonas arsenicoxydans CECT 7543	47.6
	Pseudomonas mandelii LMG 21607	47.9
	Pseudomonas mucidolens LMG 2223	48.7
