# Supplementary material for: Multilocus sequence based identification and adaptational strategies of Pseudomonas sp. from the supraglacial site of Sikkim Himalaya
Source: PLoS One. 2022 Jan 24;17(1):e0261178. doi: 10.1371/journal.pone.0261178 (PMC8786180; doi:10.1371/journal.pone.0261178)
Supplement: S5 Table — (PDF) [file pone.0261178.s005.pdf]

Supplementary Table S5. Genome features of *Pseudomonas* strains.

	**ERGC3:01**	**ERGC3:05**
**Attributes**		
Genome size (bp)	6,534,826	6,496,199
GC content (%)	59.64	59.65
Plasmid	0	0
DNA contigs	2	1
Total genes	6,075	6,016
Total predicted CDS	5,985	5,926
rRNAs	19	19
tRNAs	67	67
ncRNA	4	4
