# Supplementary material for: Multilocus sequence based identification and adaptational strategies of Pseudomonas sp. from the supraglacial site of Sikkim Himalaya
Source: PLoS One. 2022 Jan 24;17(1):e0261178. doi: 10.1371/journal.pone.0261178 (PMC8786180; doi:10.1371/journal.pone.0261178)
Supplement: S1 Fig — (PDF) [file pone.0261178.s006.pdf]

Figures

Supplementary Figure S1

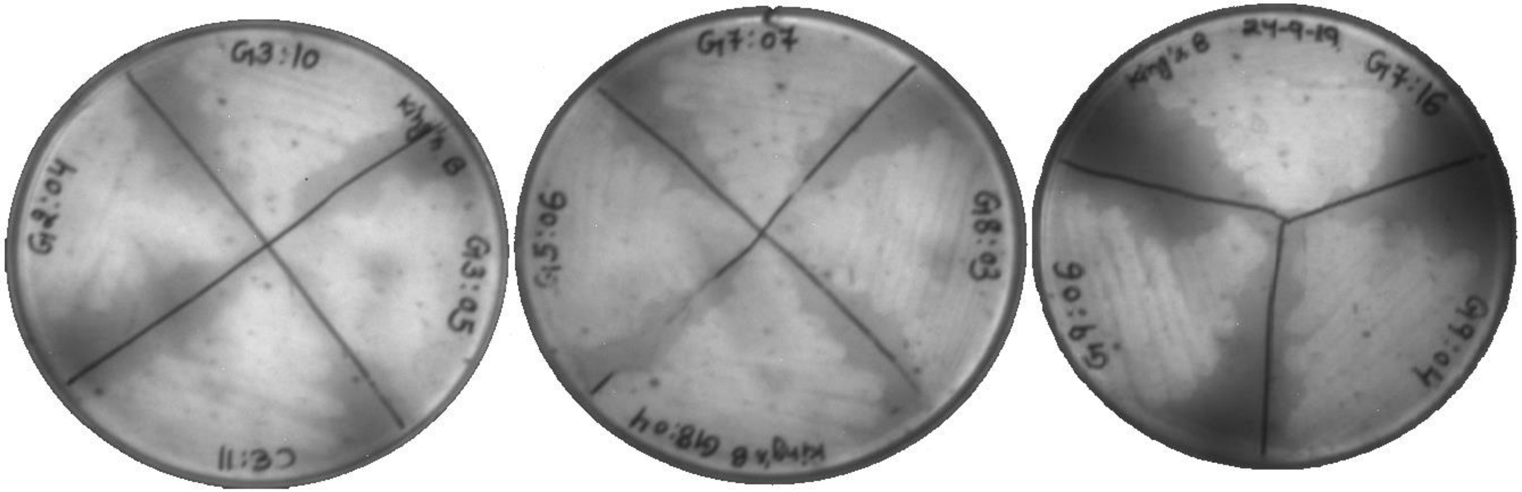

Fig. S1 *Pseudomonas* strains in King's B media showing fluorescence under UV light
