# Supplementary material for: Multilocus sequence based identification and adaptational strategies of Pseudomonas sp. from the supraglacial site of Sikkim Himalaya
Source: PLoS One. 2022 Jan 24;17(1):e0261178. doi: 10.1371/journal.pone.0261178 (PMC8786180; doi:10.1371/journal.pone.0261178)
Supplement: S2 Fig — Sequences of the neighbouring strains were retrieved from the complete genome sequences from GenBank database. Support values are calculated from 500 rapid bootstrap replicates. BT values of 50 or more are indicated at branching points. The scale bar corresponds to the average number of nucleotide substitutions per site. Our test strains are marked in bold. E. coli K12 MG1655 was used as the outgroup organism. Partitionfinder v2.1.1 was used to determine the best-fit partitioning schemes and substitution models of molecular evolution. (PDF) [file pone.0261178.s007.pdf]

Supplementary figure S2

a. RAxML tree based on *GyrB* gene

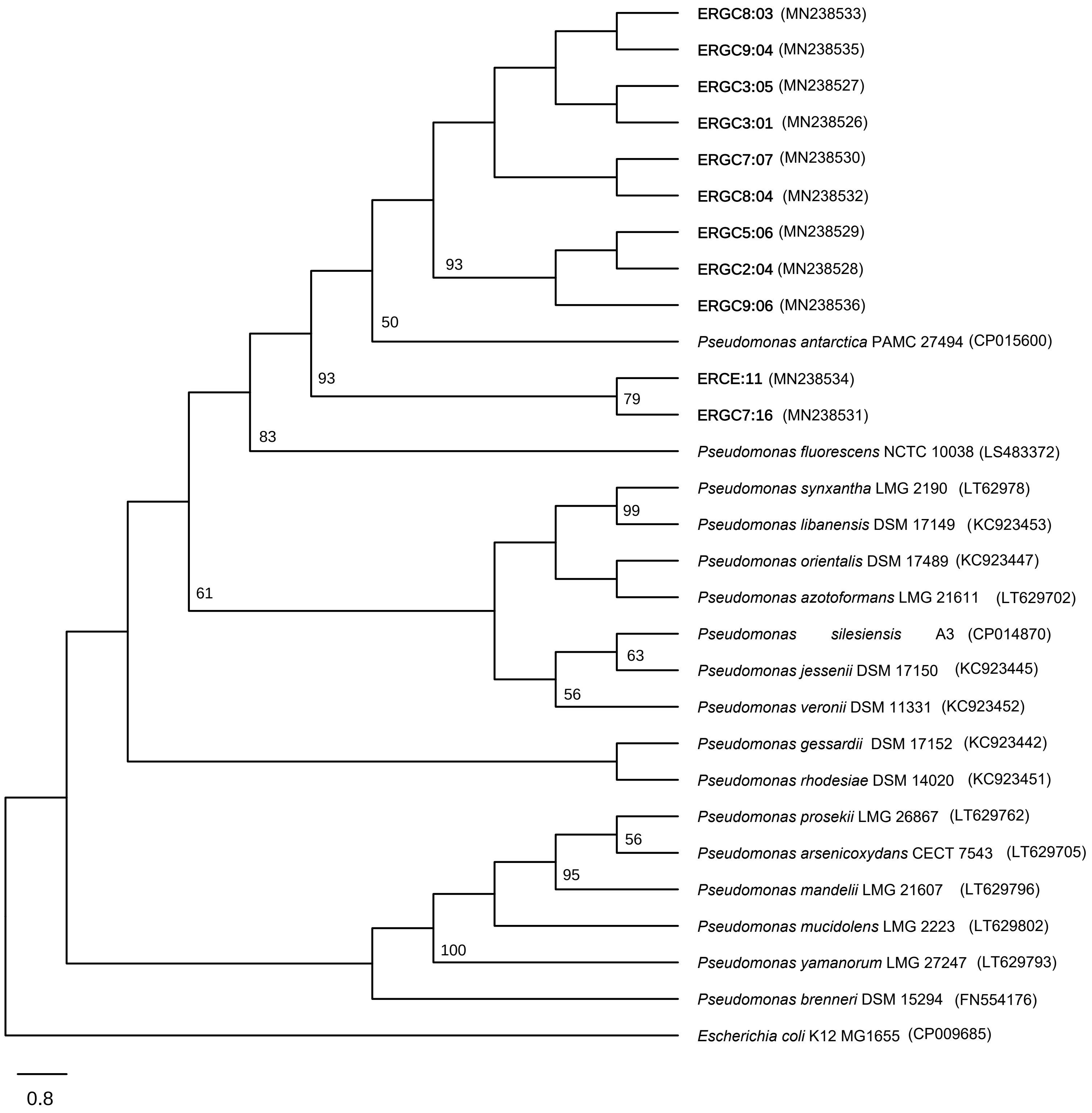

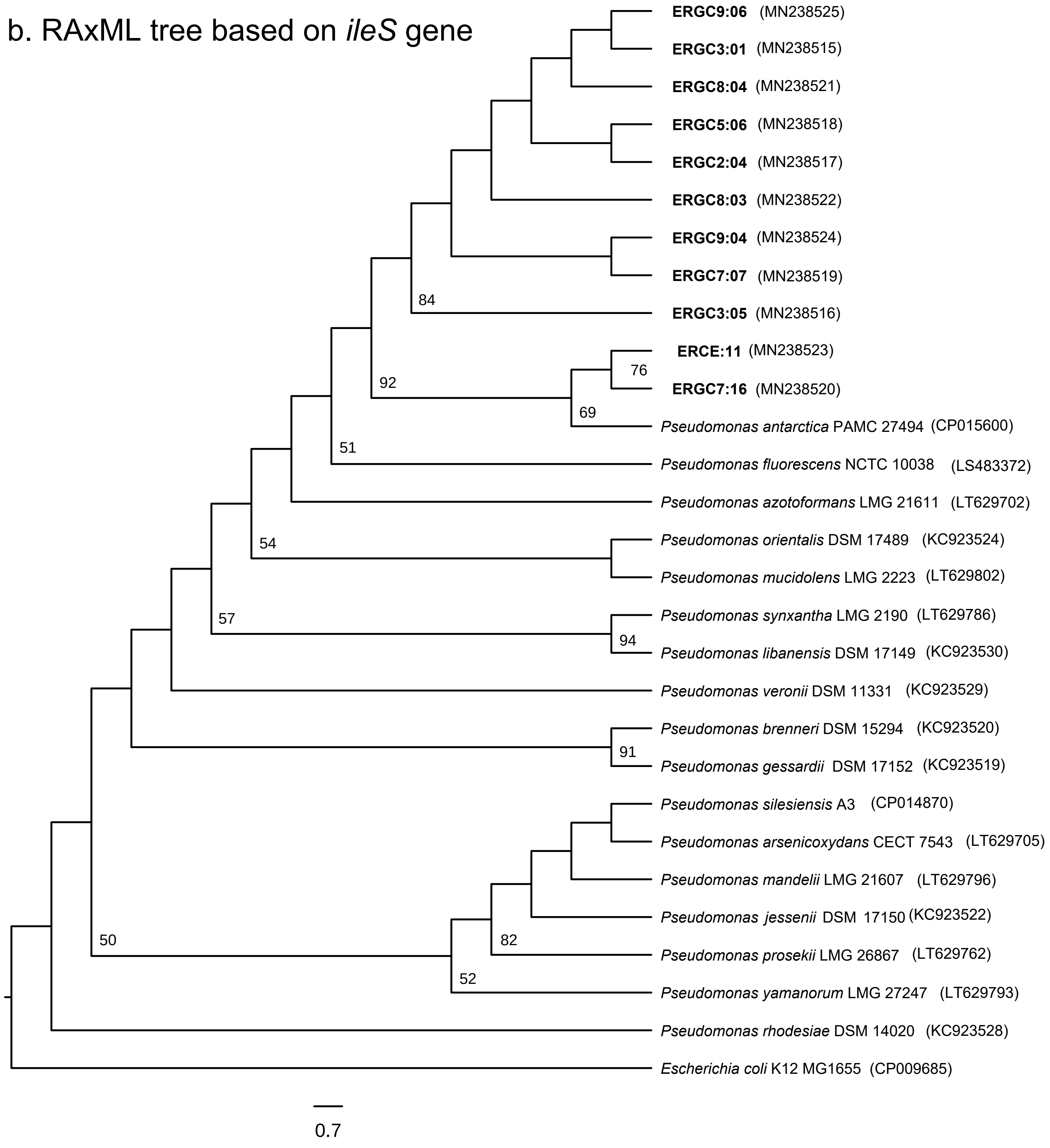
b. RAxML tree based on *ileS* gene
ERGC9:06 (MN238525)
ERGC3:01 (MN238515)
ERGC8:04 (MN238521)
ERGC5:06 (MN238518)
ERGC2:04 (MN238517)
ERGC8:03 (MN238522)
ERGC9:04 (MN238524)
ERGC7:07 (MN238519)
84
ERGC3:05 (MN238516)
ERCE:11 (MN238523)
76
92
ERGC7:16 (MN238520)
69
Pseudomonas antarctica PAMC 27494 (CP015600)
51
Pseudomonas fluorescens NCTC 10038 (LS483372)
Pseudomonas azotoformans LMG 21611 (LT629702)
Pseudomonas orientalis DSM 17489 (KC923524)
54
Pseudomonas mucidolens LMG 2223 (LT629802)
Pseudomonas synxantha LMG 2190 (LT629786)
57
94
Pseudomonas libanensis DSM 17149 (KC923530)
Pseudomonas veronii DSM 11331 (KC923529)
Pseudomonas brenneri DSM 15294 (KC923520)
91
Pseudomonas gessardii DSM 17152 (KC923519)
Pseudomonas silesiensis A3 (CP014870)
Pseudomonas arsenicoxydans CECT 7543 (LT629705)
Pseudomonas mandelii LMG 21607 (LT629796)
Pseudomonas jessenii DSM 17150 (KC923522)
50
82
Pseudomonas prosekii LMG 26867 (LT629762)
52
Pseudomonas yamanorum LMG 27247 (LT629793)
Pseudomonas rhodesiae DSM 14020 (KC923528)
Escherichia coli K12 MG1655 (CP009685)
0.7

c. RAxML tree based on *nuoD* gene

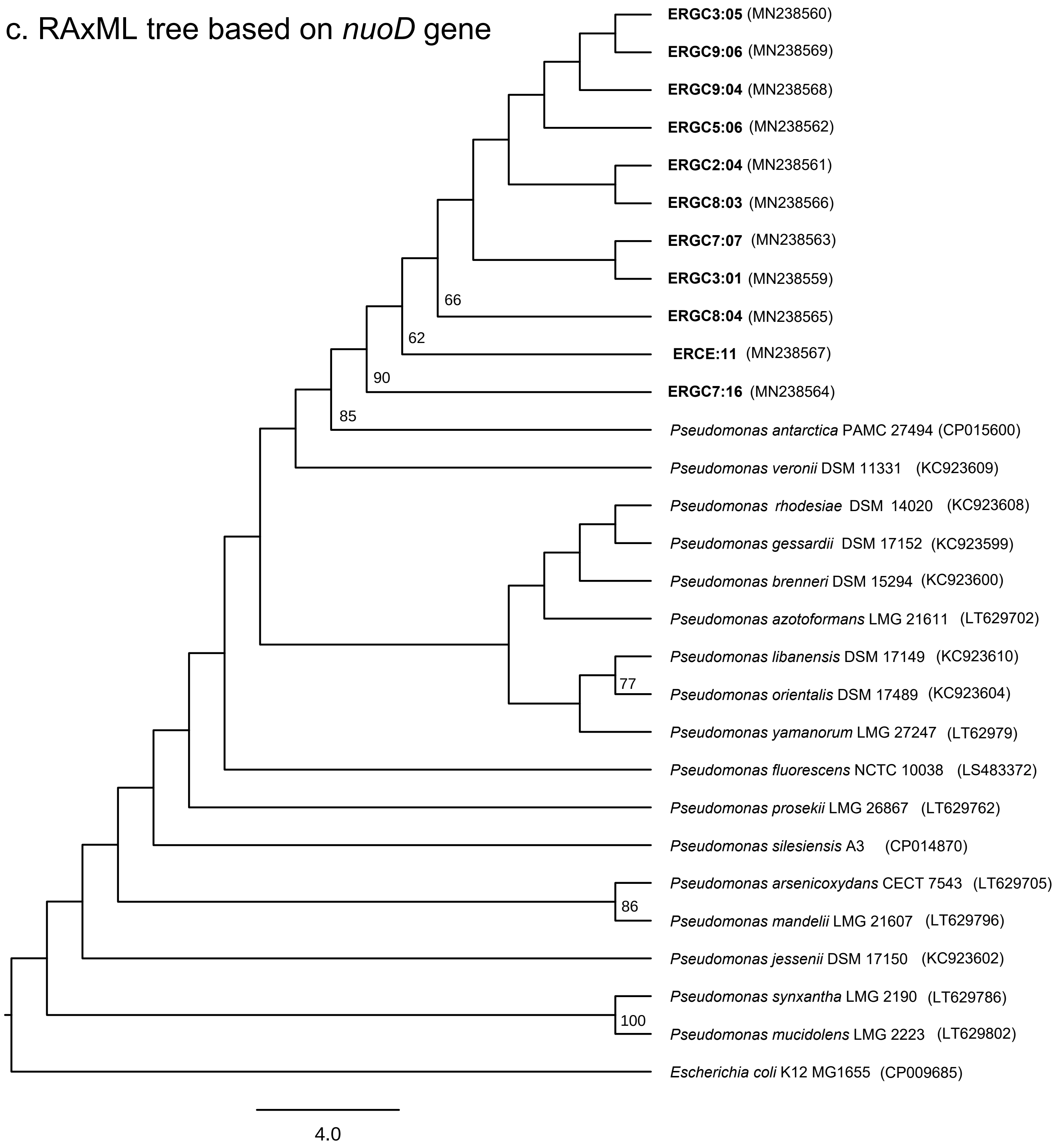

d. RAxML tree based on *recA* gene

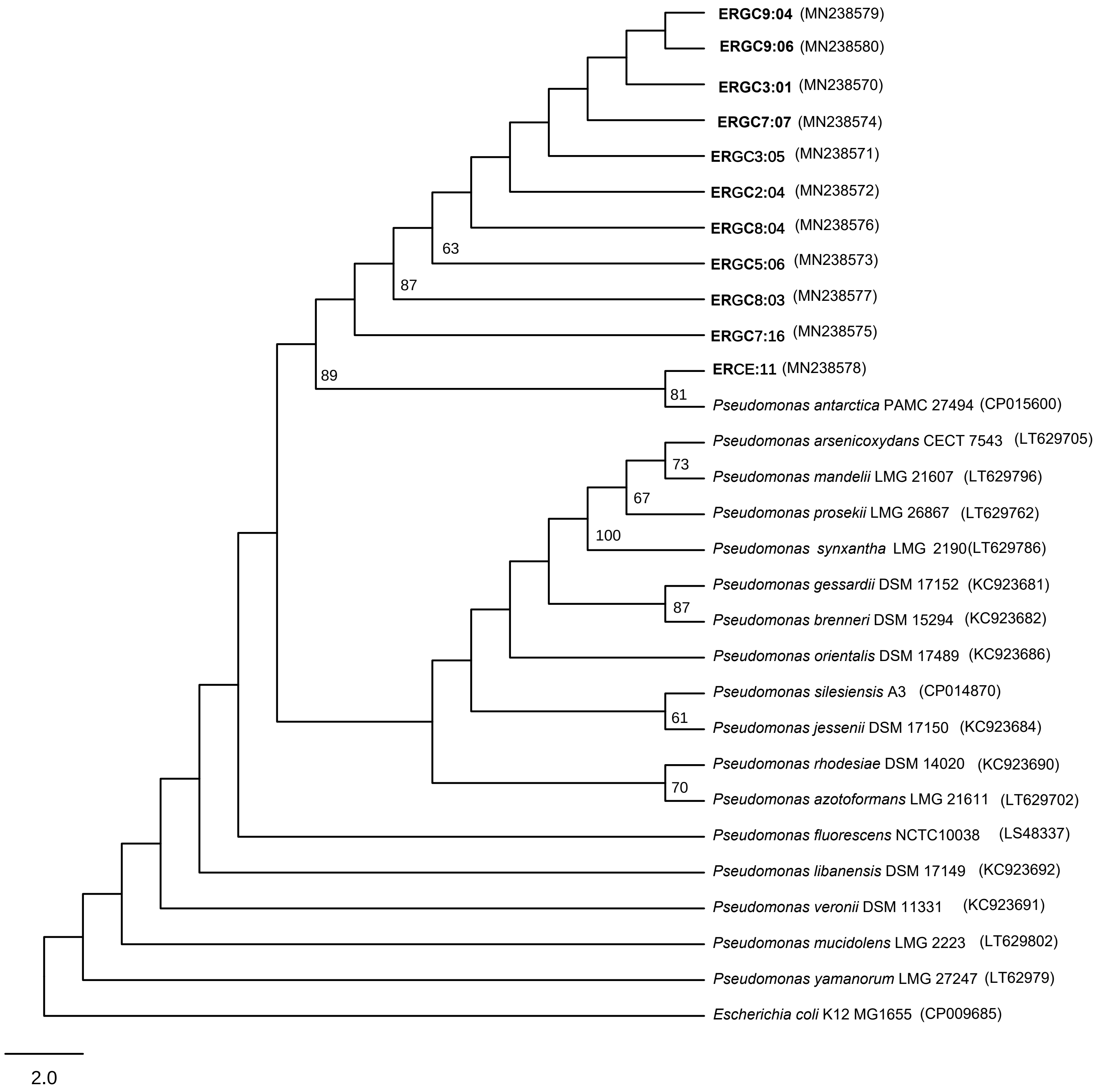

e. RAxML tree based on *rpoD* gene

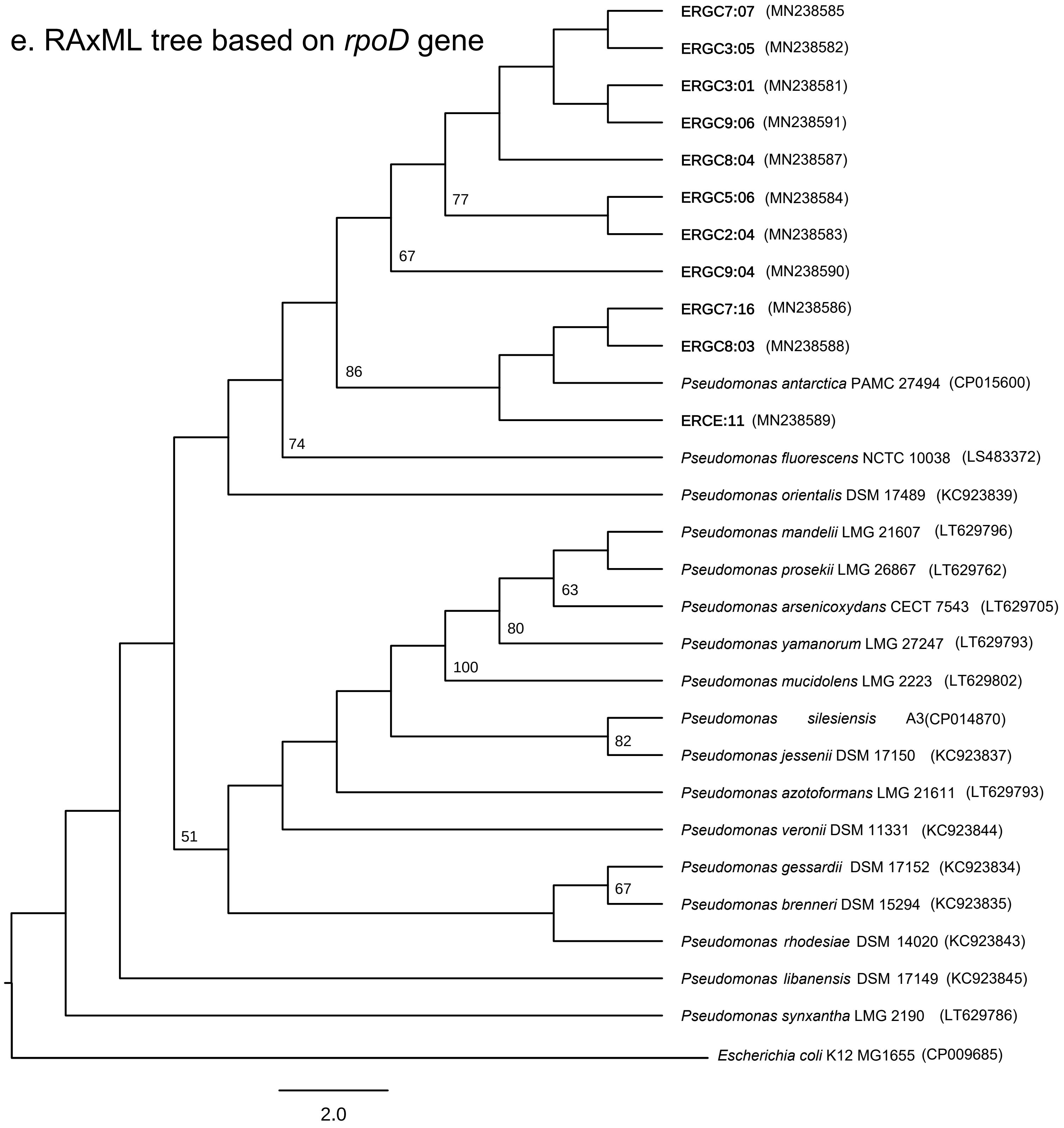

Supplementary Figure S2. Phylogenetic trees of 11 *Pseudomonas* strains based on individual protein-coding genes *gyrB*, *ileS*, *nuoD*, *recA*, and *rpoD* using RAxML. Sequences of the neighbouring strains were retrieved from the complete genome sequences from GenBank database. Support values are calculated from 500 rapid bootstrap replicates. BT values of 50 or more are indicated at branching points. The scale bar corresponds to the average number of nucleotide substitutions per site. Our test strains are marked in bold. *E. coli* K12 MG1655 was used as the outgroup organism. Partitionfinder v2.1.1 was used to determine the best-fit partitioning schemes and substitution models of molecular evolution.
