# Supplementary material for: Multilocus sequence based identification and adaptational strategies of Pseudomonas sp. from the supraglacial site of Sikkim Himalaya
Source: PLoS One. 2022 Jan 24;17(1):e0261178. doi: 10.1371/journal.pone.0261178 (PMC8786180; doi:10.1371/journal.pone.0261178)
Supplement: S4 Fig — Circular genome representations of Pseudomonas sp. (A) ERGC3:01 and, (B) ERGC3:05, created using CGView v2.0.2. (PDF) [file pone.0261178.s009.pdf]

Supplemenary figure S4

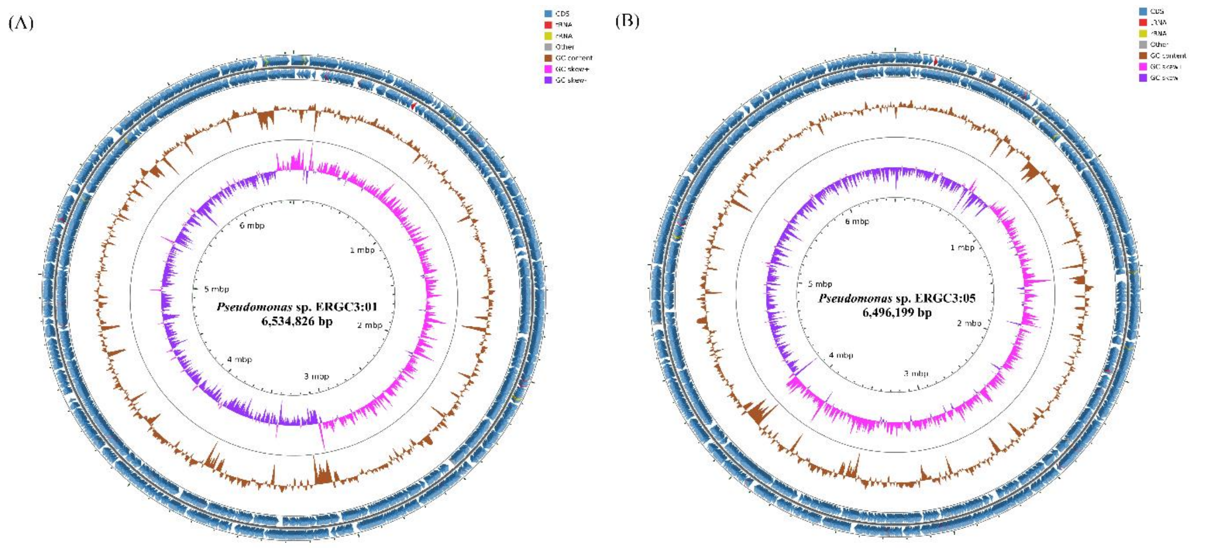

Fig. S4 Circular genome representations of *Pseudomonas* sp. (A) ERGC3:01 and, (B) ERGC3:05, created using CGView v2.0.2.
